# Deep critical zone controls on shallow landslides

**DOI:** 10.1073/pnas.2524542123

**Published:** 2026-03-18

**Authors:** Seulgi Moon, Giuseppe Formetta, Justin T. Higa, Riccardo Busti, Dino G. Bellugi, David G. Milledge, Brian A. Ebel, William E. Dietrich

**Affiliations:** ^a^Department of Earth, Planetary, and Space Science, University of California, Los Angeles, CA 90095; ^b^Department of Civil, Environmental and Mechanical Engineering, University of Trento, Trento I-38123, Italy; ^c^Department of Geography, University of California, Berkeley, CA 94720; ^d^School of Engineering, Newcastle University, Newcastle upon Tyne NE1 7RU, United Kingdom; ^e^United States Geological Survey, Water Resources Mission Area, Burlington, VT 05405; ^f^Department of Earth and Planetary Science, University of California, Berkeley, CA 94720

**Keywords:** weathering, critical zone, landslide, natural hazard, hydrology

## Abstract

The deep critical zone (CZ) has long been recognized for its importance in influencing shallow landslides but was not considered feasible to include in slope stability models at the watershed scale. Here, we demonstrate that simple approximations of the CZ in a fully coupled hydrologic and soil slope stability model can effectively capture the location, timing, and likely size of shallow landslides. To achieve this, we use coupled, process-based models that incorporate the effects of 1) deep CZ structures, 2) three-dimensional transient hydrology, and 3) multidimensional slope stability, calibrated with data from an intensively monitored field site. Our results show that the hydrologically active deep CZ guides groundwater flow, influencing where it drains from or exfiltrates to the soil mantle and producing distinct patterns of soil saturation and seepage forces at the soil–bedrock boundary. A deep conductive, weathered bedrock drains the soil mantle, reducing the likelihood of destabilizing pore pressures, while the downslope thinning of the CZ forces groundwater to the surface. This pattern creates localized instability and a tendency for similar-sized landslides across the landscape. In contrast, the absence of conductive weathered bedrock results in more widespread destabilizing pore pressures, leading to larger landslides and the likelihood of landslides earlier in a storm than in landscapes underlain by a deep CZ. Our findings suggest that first-order variations of deep CZs can provide physical explanations for variations observed in the susceptibility, magnitude, and timing of shallow landslides, and that CZ structure may be inferred from patterns and timing of landsliding.

## Significance

Shallow landslides typically involving only the soil mantle constitute both a primary process driving landscape evolution and a substantial geologic hazard. Theory and empirical documentation of shallow landslides generally assume that the elevated pore pressures that destabilize soils arise from runoff only within the soil. In many landscapes, however, it has been recognized that groundwater emerging from underlying weathered bedrock can generate destabilizing pore pressures. Here, we develop a coupled hydrologic and slope stability model that explicitly explores how a conductive bedrock weathering zone guides storm runoff and localizes shallow landslides across a watershed. The distinctiveness of the location, size, and timing of predicted landslides suggests that the influence of subsurface structure may be estimated from landslide mapping patterns.

For decades, it has been recognized that on steep soil-mantled hillslopes, storm-driven groundwater build-up in underlying weathered and fractured bedrock can lead to downslope exfiltration back into the soil, destabilize the soil mass, and generate shallow landslides, which can mobilize as destructive debris flows (e.g., refs. 1–5). The difficulty in attempting to monitor and describe the locally heterogeneous, variably weathered bedrock beneath the soil—along with the virtually unknown hydrologic properties of this subsurface material and its spatial variability across watersheds—has made it seemingly impossible to include this significant subsurface domain in models of shallow landsliding. Instead, shallow landslide models, especially for hazard warnings and land management decisions, have relied on either empirical estimation and statistical correlation (e.g., refs. 6–9) or simplified hydrologic models that ignore the subsurface hydraulic dynamics beneath the soil (e.g., refs. 10–12).

Recently, researchers have been working to observe and predict spatial variations of weathered bedrock in Earth’s near-surface layers. As bedrock is exhumed toward the surface, weathering driven by hydrobiogeochemical and mechanical processes results in a porosity that stores and transmits water, forming the base of the critical zone (CZ) (13–15). Geophysical surveys using seismic refraction tomography (16–18) show that this bedrock weathering can be highly variable, forming CZ layers below the land surface that differ across a landscape. Process-based explanations for weathering bedrock and shaping deep CZ architecture include chemical weathering in preexisting, interconnected bedrock fractures (19), frost cracking (20), reactive transport and groundwater flow (21–23), drainage due to channel incision (24), and subsurface stresses (16, 25). Comparisons of geophysical observations with process-based modeling have achieved some success in predicting the spatial variations of deep CZ structures (16–18, 24).

This permeable weathered bedrock in the deep CZs acts as groundwater storage (1, 24, 26), influences infiltration, exfiltration, and lateral subsurface flow across landscapes (1–4, 27–30), and plays an important role in shallow landslide susceptibility (e.g., ref. 31). Various studies, including theoretical analysis (32) and field observations (2, 4, 5), demonstrate the fundamental influence of bedrock permeability fields and groundwater flow paths on landslide potentials. For example, variations in weathered bedrock thickness resulting from different lithologies have been shown to influence subsurface water storage capacities, groundwater flow paths, and streamflow dynamics, even under similar average precipitation conditions (4, 33). These variations in the deep CZ can lead to substantial differences in seepage flow magnitude and direction as well as pore-water pressure development at the soil–bedrock interface, all of which likely affect the stability and characteristics of shallow landslides (3, 4). However, there is a lack of studies that incorporate groundwater dynamics within spatially varying, permeable, and weathered bedrock into slope stability models or systematically examine the impact of a variable CZ on shallow landslides.

In this study, we investigate how spatially varying weathered bedrock within the deep CZ influences the occurrence, location, size, and timing of shallow landslides. We use a fully coupled, process-based modeling framework that integrates numerical models of deep CZ structures, three-dimensional transient hydrology, and multidimensional slope stability at a watershed scale. First, we generate five theoretical CZ structures based on alternative conceptions of dominant weathering processes to explore the impact of varying CZ depth extent from a well-studied benchmark site near Coos Bay, OR. Next, we simulate transient hydrologic flow within these structures under realistic, intense rainfall conditions to estimate spatially variable soil saturation and groundwater seepage forces at the soil–bedrock boundary. Finally, we assess how first-order variations in the hydrologically active deep CZ, particularly catchment-scale patterns of CZ thickening and thinning, influence the occurrence, location, timing, and potential size of shallow landslides, offering physical insights into the large natural variations observed.

## Numerical Simulation

**Fig. 1.**
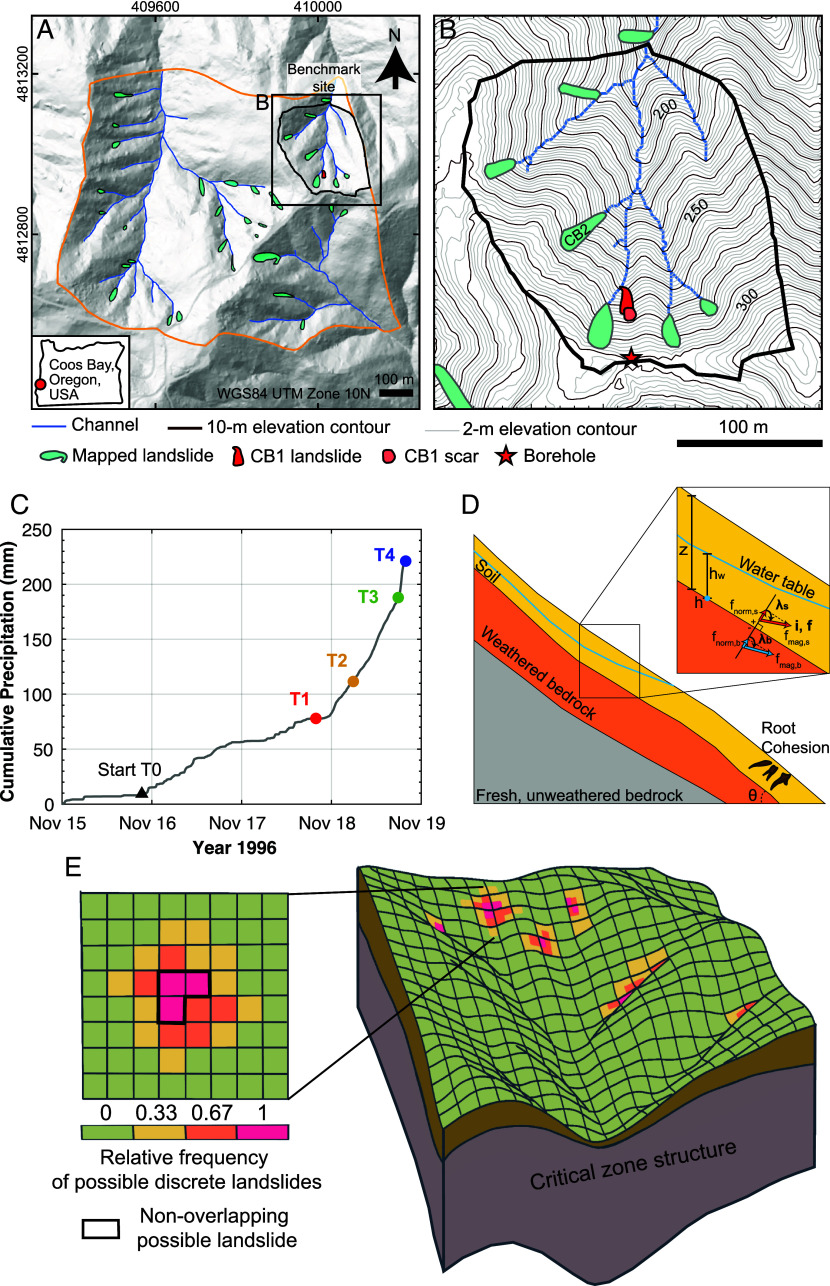
Map of the benchmark site and landslide model schematic. (*A*) Map of the benchmark site (black outline) and surrounding Mettman Ridge area (orange outline) near Coos Bay, OR (37, 38). Landslides mapped between 1987 and 1996 (36) are shown in teal polygons. (*B*) Benchmark site showing mapped landslides, the channel network, and the location of the 35 m-deep CB1 borehole. (*C*) Cumulative precipitation record of the storm event that triggered the CB1 landslide at 20:00 11/18/1996. GEOtop model output times, from T1 to T4, are labeled with corresponding colors. The onset of significant rainfall is indicated by the black triangle (T0), and the time of the CB1 landslide event is T4. (*D*) Schematic of CZ structure and hydrologic output variables that affect slope stability. Examples of seepage vectors are shown for a cell in soil (red) above and a cell in bedrock (blue) below the soil–bedrock boundary in two dimensions. *h* = pressure head at soil–bedrock boundary [m], *h_w_* = the height of water table (pressure head = 0) above the soil–bedrock boundary [m], *z* = soil thickness [m], *λ* = seepage vector orientation [deg.] in the downslope direction relative to the outward-directed vector normal to soil–bedrock boundary, *i* = seepage vector [m/m], *f =* seepage flux [m/s], *f_mag_* = magnitude of seepage flux [m/s], *f_norm_* = magnitude of seepage flux normal to soil–bedrock boundary [m/s], *θ* = slope of soil–bedrock boundary [deg.]. These terms are defined only for the cells, either soil directly above (subscript, *s*) or bedrock directly below (subscript, *b*) the soil–bedrock boundary. The brown-colored roots indicate the presence of root cohesion, which affects the entire landscape based on soil thickness. (*E*) Schematic oblique view of the benchmark site looking roughly southward (*Right*) and the surface discretized into cells (*Left*). Cells are colored based on the relative frequency of potential discrete landslides, calculated as the frequency with which a given cell is included in unstable cell clusters, normalized by the total number of possible landslides (*n* = 3 in this example). An example of a nonoverlapping pruned possible landslide is outlined in black. Figure modified from Bellugi et al. (38).

We utilize detailed subsurface observations, hydrologic data, and landslide mapping from previous studies in Coos Bay, OR, to calibrate weathering, hydrologic, and slope stability models (Fig. 1 *A* and *B* and *SI Appendix*, section 1) (1, 2, 27, 28, 34–36). Detailed explanations for the models are presented in *Materials and Methods* and *SI Appendix*, section 2. Below, we briefly describe our numerical simulations for CZs, hydrology, and slope stability.

We set a goal to model how the predicted CZ structure at a catchment scale, beyond a single hillslope, varies across ridge and valley topography and, in turn, how such structures influence predicted patterns of shallow landslides. To do this, we simulate deep CZ development that represents plausible, three-dimensional variations at the benchmark site with an area of 0.04 km^2^ (Fig. 1*A*). Five theoretical CZ structures based on different assumptions of underlying bedrock properties and structures are explored. Three models capture the observed first-order variations in weathered bedrock thickness, which is thick under ridges and thin or absent near valleys, calibrated using field measurements. We compare these CZ structures to those with no underlying conductive weathered bedrock. By testing five alternative deep CZ structures, we identify the sensitivity of shallow landslide susceptibility and characteristics to the presence and shape of weathered bedrock in deep CZs.

The CZ depth profile consists of soil at the top, variably developed weathered bedrock in the middle, and unweathered bedrock at the bottom. The hydrologic properties of the CZ layers are derived from field data (*SI Appendix*, Tables S1 and S2) (1, 27, 28, 34). We followed the approach of Ebel et al. (30), who used the hydrologic properties for the soil based on the field observations at CB1 (1, 28, 30, 34, 35) and those for weathered and unweathered rocks based on field measurements from another site (i.e., Yucca Mountain, Nevada). We model the production and transport of soil to predict the spatial variation in soil thickness mantling the landscape (*SI Appendix*, section 2.1). CZ(soil) and CZ(soil, low K) represent the thinnest CZ, consisting of a soil layer without weathered bedrock. For these cases, we assigned different bedrock hydraulic conductivities to the unweathered bedrock (i.e., 5.0 × 10^−7^ m/s for permeable bedrock beneath CZ(soil) and 5.0 × 10^−12^ m/s for impermeable bedrock beneath CZ(soil, lowK). We note, however, the hydraulic conductivity of 5.0 × 10^−7^ m/s is likely higher for fresh, unweathered sandstone bedrock at our site (24, 31). In the model, we applied impermeable lateral boundaries and an impermeable bottom boundary located 40 m below the topography surface, except at the catchment outlet, where we allow a top 24 m free-flux boundary (*SI Appendix*, section 2.2). The bottom boundary has the same topography as the ground surface. The choice of a 40-m model depth is guided by local field observations from a 35-m CB1 borehole core (35), which shows minimal chemical weathering in samples and fractures that were cemented and closed (enabling complete core sample recovery) collected from depths of 30 to 35 m.

The spatial variation of weathered bedrock thickness, characterized by a higher bedrock conductivity of 7.2 × 10^−5^ m/s, is modeled using two alternative weathering models: the bedrock drainage model (24) for CZ(RD4m) and CZ(RD9m), and the topographic stress model (16, 25) for CZ(stress). Our models are calibrated using field observations of deep, weathered rock properties at this site (35) (*SI Appendix*, Table S3 and Figs. S1 and S2). The weathered bedrock depth and subsurface water storage below the soil generally increases from CZ(RD4m), CZ(RD9m), to CZ(stress). However, the hydraulic conductivity of unweathered bedrock and the soil mantle in these cases remain constant at 5.0 × 10^−7^ and 3.4 × 10^−4^ m/s, respectively.

We use the hydrologic model GEOtop 2.0 (39) to simulate variably saturated water fluxes through CZ structures in three dimensions. The results of the hydrologic model were inputs to the slope stability model to predict the pore pressures and seepage forces at the soil–bedrock boundary. We use the documented natural rainfall record that triggered CB1 landslide in 1996 (hereafter, CB1 storm) (Fig. 1*C*) (*SI Appendix*, section 1). Following a previous study at the site (30), we use a 2-mo warm-up period for the model, which was initiated at 00:00 13 September 1996 and ran to 00:00 15 November when significant continuous rainfall began. We recorded hydrologic model outputs at 10-min intervals, using a 10-min time step. We set a hydrologic initial condition of the pressure head at every subsurface node based on a groundwater table 2 m below the surface. The details of hydrologic model parameterization, initial and boundary conditions, rainfall input records, and model output postprocessing are provided in *SI Appendix*, section 2.2.

The onset of a continuous, significant rainfall that induced the CB1 landslide started around 21:30 15 November 1996 (T0, Fig. 1*C*). We examined hydrologic model outputs (recording results at 10-min intervals) at four different timesteps after the initiation of rainfall: 17 November 1996, 20:00; 18 November 1996, 06:00; 18:00; and 20:00 (hereafter, T1, T2, T3, and T4; Fig. 1*C* and *SI Appendix*, section 2.2). These times correspond to 24, 14, 2, and 0 h before the CB1 landslide. The corresponding durations after T0 for these times correspond to 46.5, 56.5, 68.5, and 70.5 h. The measured rainfall intensities for those durations correspond to 1.47, 1.80, 2.60, and 3.00 mm/h. The 24-h-averaged rainfall intensities leading to the four timesteps increased with time from 1.01, 2.37, 5.00, and 5.86 mm/h (see Fig. 1*C*). The intensity (*I,* mm/h) and duration (*D,* h) of rainfall events that triggered shallow landslides are estimated as *I* = 9.9*D*^−0.52^, based on 35 landslides between 1987 and 1998 from the Mettman Ridge area including our benchmark site (36). Our simulations from T2 to T4 likely represent rainfall intensity conditions that generate the landslides in this study area (*SI Appendix*, section 2.2).

For these four timesteps, we calculate the spatially varying saturation ratio *h/z* [m/m] as a ratio of pressure head at the soil–bedrock boundary *h* [m] to soil thickness *z* [m]. To document exfiltration or infiltration across the soil–bedrock boundary, we calculate several hydrologic quantities from soil above and bedrock below the soil–bedrock boundary, which are denoted with subscripts s and b, respectively (Fig. 1*D*). We calculate the hydraulic gradient vector (hereafter, the seepage vector *i*) specifically at the soil–bedrock boundary [adopting the approach of Iverson and Major (40)], recording the magnitude *i_mag_* [m/m] and angular orientation *λ* [deg.]. The *λ* [deg.] is measured in the downslope direction relative to the outward-directed vector normal to the soil–bedrock boundary surface. A *λ* value of 90° or 0° [i.e., cos(*λ*) of 0 and 1] indicates surface parallel or surface normal exfiltrating seepage fluxes, respectively. We also calculate the specific discharge vector (hereafter, seepage flux *f* [m/s]) as the negative product between hydraulic conductivity *K* and seepage vector *i*. Its magnitude is *f_mag_*[m/s], and its component normal to the soil–bedrock boundary is denoted as *f_norm_* [m/s]. We calculate the ratio of *f_mag_* in bedrock to *f_mag_* in soil (hereafter, *f_mag,b_*/*f_mag,s_*) and the ratio of *f_norm_* in bedrock to *f_mag_* in soil (hereafter, *f_norm,b_*/*f_mag,s_*) to assess the magnitude of seepage flux from the bedrock relative to seepage flux in soil (*SI Appendix*, section 2.3, see *SI Appendix*, Table S4 for description of variables).

Last, we adapt and modify a multidimensional slope stability model coupled with a spectral search algorithm to predict the stability of clusters of adjacent grid cells (Fig. 1*E*) (37, 38, 41, 42) (*SI Appendix*, section 2.3). Following the methods described by Bellugi et al. (38), we use modeled soil thickness and cohesion parameters. In addition, we modify force balance equations therein to incorporate spatially variable seepage forces from our hydrologic model, aligning with the formulation by Iverson and Major (40).

Our model identifies clusters of adjacent grid cells of soil that are unstable (i.e., a factor of safety *FoS* < 1) (37, 38) and generates maps that show all identified unstable clusters, many of which overlap in space. By incorporating hydrologic outputs from the given time steps (T1–T4), we identify thousands of possible configurations (with significant overlap) across a wide range of *FoS*. Hereafter, we refer to these overlapping, unstable cell clusters as possible discrete landslides (Fig. 1*E*). For each possible discrete landslide, we calculate its *FoS*, size, and median topographic index [defined as the median of the log_10_-transformed values of the ratio between drainage area and cell width multiplied by the sin of slope angle, a/(**b*∙*sinθ), for all cells within the slide (43)]. This index serves as a metric for landslide position, with low values in steep nonconvergent areas and high values in the valleys (*SI Appendix*, section 2.3). The relative frequency of possible discrete landslides is calculated as the fraction of its count for each cell relative to the total number identified across the study area (Fig. 1*E*).

We record the total predicted unstable area based on relative frequency greater than zero (hereafter, nonzero relative frequency) in the model domain, the spatial distribution of the relative frequency of all possible discrete landslides, the distributions of landslide sizes, and the distributions of topographic indices at the specified times. These results allow us to examine how the occurrence, relative frequency, size, location, and timing of shallow landslides evolve during a rainstorm and vary based on CZ structures in a statistically representative way. In addition, we show the spatial distributions of nonoverlapping single discrete landslides. To estimate the location of single discrete landslides, we use the pruning method developed by Bellugi et al (38) that selects those nonoverlapping collections of unstable cells with the minimum *FoS* (hereafter, pruned landslide). We compare our model results with seven mapped landslides from our benchmark site (36) (*Materials and Methods*).

## Results

### Modeled CZ Structures.

**Fig. 2.**
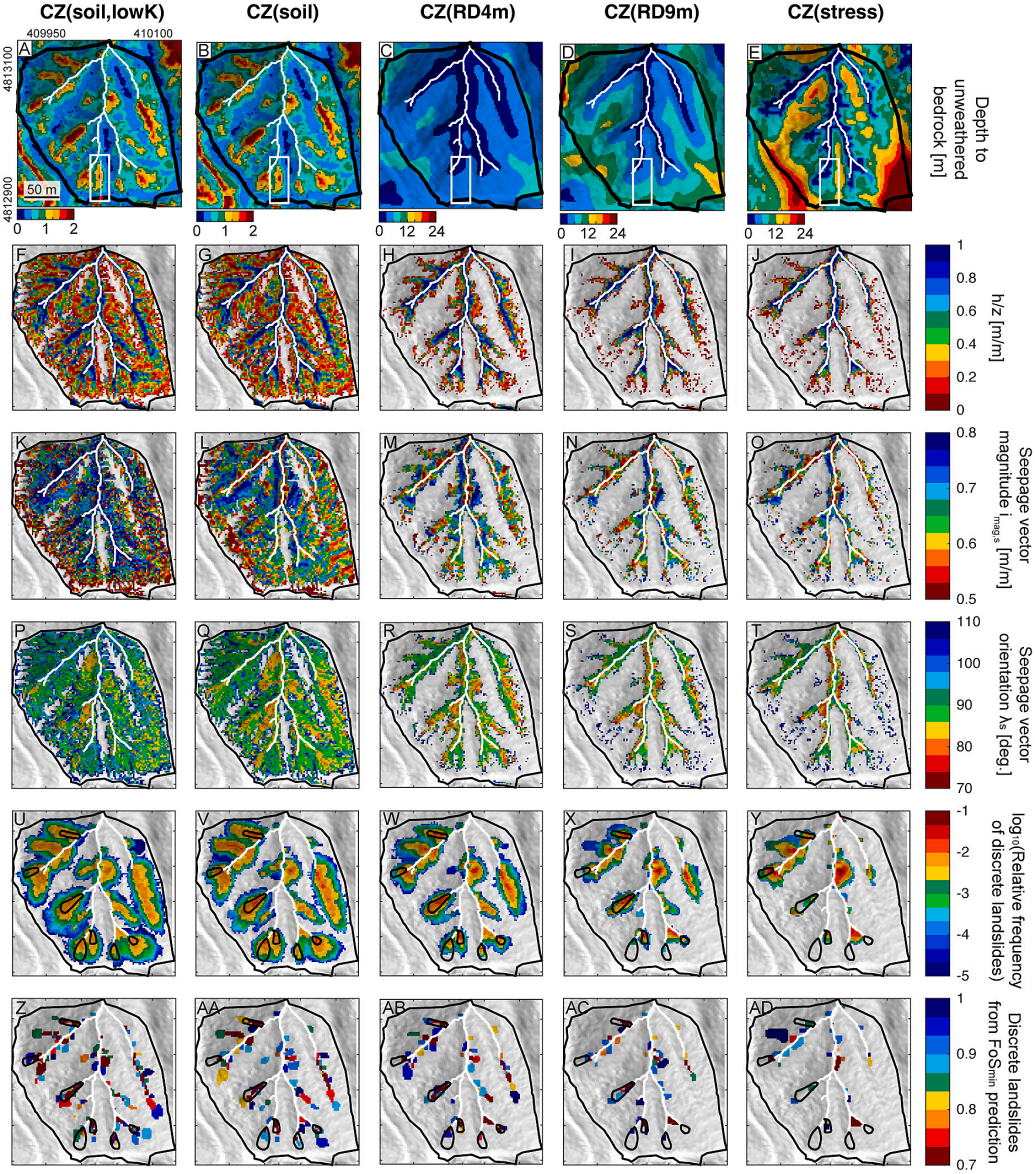
Depth to unweathered bedrock, soil saturation, seepage, and potential landslides for different CZ models at the time of the CB1 landslide. (*A*–*E*) Map of depth to unweathered bedrock for different CZ models, (*F–J*) Soil saturation *h/z*, the ratio of pressure head at the soil–bedrock boundary *h* and soil thickness *z*, (*K*–*O*) seepage vector magnitude *i_mag,s_*, and (*P*–*T*) seepage vector orientation *λ_s_* in the soil above the soil–bedrock boundary. Seepage vector orientation *λ_s_* in the downslope direction relative to the vector normal to the soil–bedrock boundary, hence 90 degrees is parallel to the boundary, with greater than infiltrating and less than exfiltrating from the bedrock. (*U*–*Y*) log_10_ of the relative frequency of possible discrete landslides, calculated as the frequency of possible discrete landslides including a specific cell normalized by the total number of possible landslides. (*Z*–*AD*) Nonoverlapping possible discrete landslides predicted by a minimum factor of safety (*FoS_min_*). The thin white line in *A* indicates the *N*–*S* transect location of Fig. 3, with a red dot at the southern end representing the CB1 borehole. The white box in *A*–*E* is centered on the location of the 1996 CB1 failure area (Fig. 1*B*) shown in *SI Appendix*, Fig. S3. Channel networks are shown as white lines, and benchmark site outline is in thick black. Mapped landslides (36) are shown in black polygons in *U*–*AD*. Projection: WGS84 UTM Zone 10 *N*.

All five CZ structures use the same modeled spatial distribution of soil thickness, which varies from 0 to 3 m-deep (Fig. 2*A*) with a mean of ~0.6 m in our benchmark site (*SI Appendix*, Fig. S3*A* and Table S3). The thickest soils are in topographic convergent zones (i.e., hollows), mainly at the heads of mapped channels, and are mostly ~2 m deep. At the CB1 landslide, the modeled soil thickness is >2 m-deep where the corresponding scar was mapped (*SI Appendix*, Fig. S3*E*), which then decreases away from the scar both upslope and downslope. We assume that stream flow can evacuate all soils transported into them. The channels are essentially devoid of soils, with a minimal soil thickness of 0.02 m therein for all scenarios. Channel head locations are based on field mapping (36).

Modeled weathered bedrock in the deep CZ shows large spatial variations and different magnitudes of thickness depending on the CZ structures (Fig. 2 *A*–*E* and *SI Appendix*, Fig. S3 and Table S3). CZ(soil, lowK) and CZ(soil) are the thinnest CZs, with soil directly on top of unweathered bedrock (Fig. 2*A*). CZ(RD4m) has a layer of weathered bedrock that increases in thickness away from channels and is ~4.5 m-deep at the CB1 borehole (Figs. 1*B* and 2*C*). CZ(RD9m) has a similar spatial pattern to CZ(RD4m) because both are based on the ratio of the unweathered bedrock surface relief, *Z_b_*, to the ground surface relief, *Z_s_*. However, weathered bedrock in CZ(RD9m) is approximately double the thickness of CZ(RD4m), at ~9 m-deep at the CB1 borehole (Fig. 2*D*). Hence, CZ(RD4m) and CZ(RD9m) predict that the thickness of weathered bedrock thins toward the channel where fresh bedrock is exposed, but CZ(RD9m) is over twice as thick at the ridge. The stress-driven structure CZ(stress) has the thickest weathered bedrock layer, up to 40 m-deep at the southeast ridge (Fig. 2*E*). Consequently, CZ(stress) has the widest range of weathered bedrock thicknesses, with bedrock at the surface near channels (i.e., 0 m-deep) that deepens toward ridges, including secondary ridges of lower relief. All three CZ structures produce spatial variations in bedrock architecture associated with three-dimensional topography (Fig. 2 *C*–*E* and *SI Appendix*, Fig. S3 *F*–*H*). At the location of the CB1 scar, CZ(RD9m) and CZ(stress) show similar shallowing patterns of unweathered bedrock. However, their patterns differ substantially elsewhere.

### Modeled Three-Dimensional Transient Hydrology.

#### Seepage flux within the CZ of the CB1 hillslope cross-section.

**Fig. 3.**
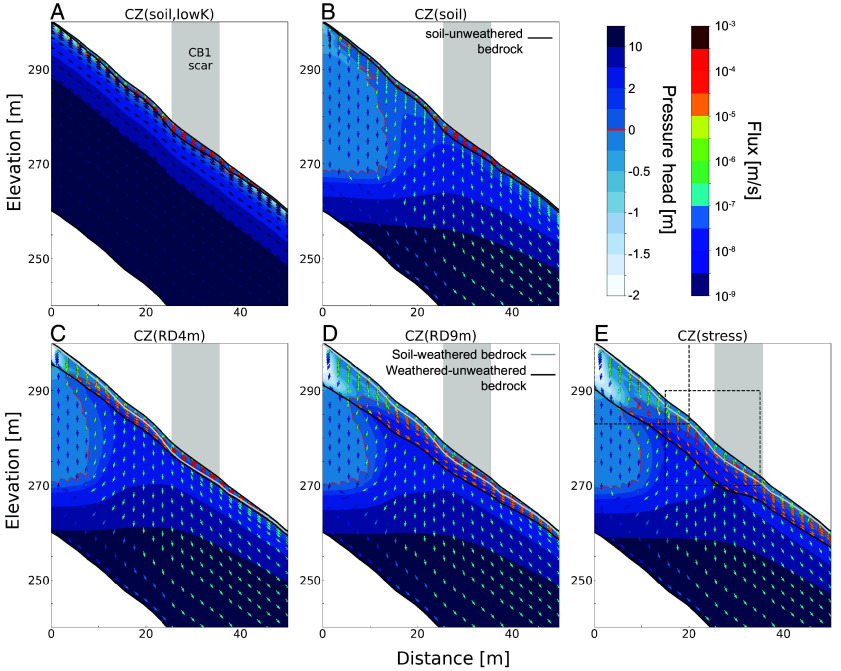
Groundwater flux magnitudes and directions for a north–south cross-section near CB1. (*A*) CZ(soil, lowK), (*B*) CZ(soil), (*C*) CZ(RD4m), (*D*) CZ(RD9m), and (*E*) CZ(stress) model scenario results at the time of the CB1 landslide. The direction and color of the arrows represent the direction and magnitude of seepage fluxes, respectively. Pressure head values are shown in the background blue hues. The extent of the CB1 scar is represented by a gray area. Fig. 2*A* and *SI Appendix*, Fig. S3 for the location of the cross-section. Red line defines the points of the domain at zero pressure head, therefore the boundary between saturated and unsaturated flow. The zoom-in views for the dashed boxes in *E* are provided in *SI Appendix*, Fig. S5.

We present how seepage flux magnitudes and directions change throughout the CB1 storm within CZ layers for different CZ structures in a two-dimensional, north–south cross-section along the axis of a subtle hollow that includes the CB1 landslide (Fig. 3 and *SI Appendix*, Figs. S4 and S5, with the location shown in Fig. 2 *A*–*E* and *SI Appendix*, Fig. S3 *E*–*H*). The first row in *SI Appendix*, Fig. S4 shows relative saturation and flow vectors after rainfall and drainage from 14 September to 17 November 1996 (T1 in Fig. 1*C*). In CZ(soil, lowK), flow in the bedrock is everywhere parallel to the ground surface and underlying parallel no-flow boundary. By contrast, in other CZ structures, the subsurface tends to be less saturated overall. The distribution and extent of saturated zones vary across scenarios, reflecting differences in CZ architecture and hydraulic properties. As the CB1 storm progresses (rows in *SI Appendix*, Fig. S4), the flux magnitudes and orientations change in response to the increase in pore pressure and saturation states within CZ layers. In the three models with a weathered bedrock layer, saturation first forms at the weathered–unweathered bedrock interface or soil–unweathered bedrock interface. As it deepens and spreads upslope, the groundwater table locally rises into the overlying soil. In particular, the lower parts of hillslopes become more saturated over time and propagate upslope (*SI Appendix*, Fig. S4).

The most upslope area under the ridge, which is underlain by thin soil and permeable unweathered bedrock, is unsaturated to considerable depth. This is consistent with field measurements (31), which recorded the groundwater table as much as ~19 m below the surface in the deep well at the ridge top in February 1992. Here, the local infiltrating rainwater is not sufficient to overcome deep groundwater drainage that is then forced laterally downslope by a lower no-flow boundary condition and no-flow boundary at the divide. Further downslope, the infiltrating water and deep drainage in the permeable bedrock are sufficient to raise the groundwater table into the weathered bedrock and soil mantle.

Depending on the CZ structure, the degree and area of saturation within the CB1 profiles differ considerably at the time of the CB1 landslide (Fig. 3, zoom-in areas in *SI Appendix*, Fig. S5). In CZ structures without weathered bedrock, all or nearly all the runoff is conducted through the soil (Fig. 3 *A* and *B*). In CZ(soil, lowK), a perched water table develops within the soil above unweathered bedrock due to the very low conductivity. The underlying bedrock remains unsaturated near the soil–bedrock boundary and does not change from the initial condition during our simulations (Fig. 3*A* and *SI Appendix*, Fig. S5 *A* and *F*). In CZ structures with permeable bedrock (Fig. 3 *B*–*E* and *SI Appendix*, Fig. S5 *B*–*E* and *G*–*J*), the seepage flux magnitudes and orientations show large variations depending on the configurations and saturation states of CZ layers and relatively small-scale variations in the topography of the weathered bedrock to fresh bedrock boundary. In unsaturated soil, weathered bedrock, and unweathered bedrock, flux vectors tend to be vertically downward oriented. Flux vectors in the saturated soil, saturated weathered bedrock, and no-flow bottom boundary are subparallel along the sloping interfaces of either the soil-weathered bedrock, the weathered–unweathered bedrock boundary, or the hydrologic model boundary.

#### Spatial distribution of soil saturation and seepage flux.

The spatial extent of the partially to fully saturated soil columns (*h/z* > 0) differs across CZ structures in all timesteps (*SI Appendix*, Fig. S6 *A*–*T*), with large differences observed at the time of the CB1 landslide (Fig. 2 *F*–*J*). In all CZ structures, areas with convergent topography [e.g., hollows (unchanneled valleys)] tend to have high *h/z* compared to those in divergent topography (e.g., ridges). In the upper part of the hillslopes, the areas remain unsaturated (*h/z* < 0) (no colors in Fig. 2 *F*–*J*). This is likely due to local runoff infiltrating into the underlying bedrock, with some lateral unsaturated flow developing at the soil–bedrock interface or within soil, as captured by our three-dimensional transient hydrologic model.

At the time of the CB1 landslide, CZ structures with no weathered bedrock tend to have more extensive areas with *h/z* > 0 (80 to 81% for CZ structures with only soil compared to 23 to 37% for CZ structures with weathered bedrock (Fig. 2 *F*–*J* and Dataset S1). Approximately 3.5 to 6.4% of saturated areas tend to have *h/z* > 1 in all CZ structures, indicating pore water pressures higher than hydrostatic pressure at fully saturated soil with a potential for overland flow (magenta color in *SI Appendix*, Fig. S7 *A*–*E* and Dataset S1). In all of these cases, CZ(stress) is distinguished by distinctly less total area of elevated pore pressure compared to other CZ structures.

In saturated soil at the soil–bedrock boundary, the mean value of the individual seepage vector magnitude *i_mag,s_* for all CZs is similar (0.63 to 0.67) to the sine of mean topographic slope of corresponding areas [*sin(θ),* 0.64 to 0.66] (Fig. 2 *K*–*O* and *SI Appendix*, Fig. S8 *A*–*E* and Dataset S1). The mean seepage vector orientation *λ_s_* for all CZs range from 92 to 97° (Fig. 2 *P*–*T* and *SI Appendix*, Fig. S8 *F*–*J*). These values are consistent with the expected hydraulic gradient magnitude [*sin(θ)*] and orientation (90°) of surface-parallel seepage flow. In mostly isolated areas of the upper hillslope, seepage fluxes in soil tend to be directed downward (*λ_s_* of 90 to 120°), indicating infiltration into the underlying bedrock. For CZ structures with weathered bedrock, these partly saturated soils with infiltration-dominated flow (*λ_s_* > 100°) appear as small patchy areas in hillslopes, where topographic convergences lead to relatively thick soils and flow accumulation. In areas close to the base of hillslopes bordering channels and hollow floors, exfiltrating seepage vectors become more horizontal (*λ_s_* < 90°) and the seepage magnitudes are high (e.g., Fig. 2 *M*–*O* and *R–T*). These patterns are especially pronounced in CZ(stress), where weathered bedrock thins considerably near channels (Fig. 2 *E* and *T*).

**Fig. 4.**
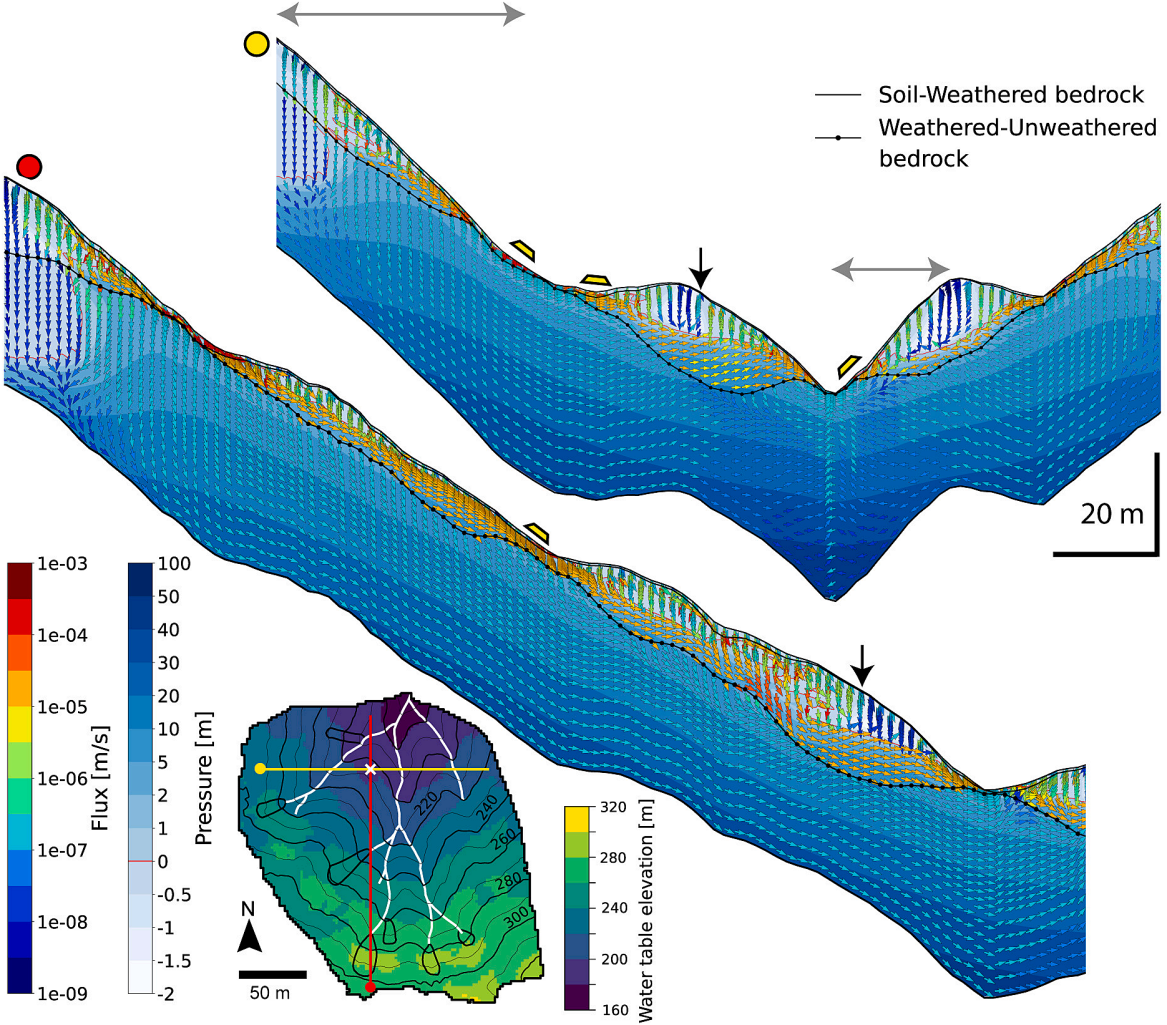
Groundwater flux magnitudes and directions for cross-sections from CZ(stress) model. Results correspond to the time of the CB1 landslide event (T4). See the inset map for the locations of the south-to-north and east-to-west cross-sections, marked in red and yellow, respectively. The intersection point is indicated by a white cross on the inset map and black arrows on the cross-sections. The inset map color indicates water table elevation, while contours represent ground surface elevation. The direction and color of the arrows indicate the direction and magnitude of seepage fluxes, respectively, within the two-dimensional cross-sectional planes derived from three-dimensional model results. Pressure head values are shown in the background. Flux magnitudes and orientations within the weathered bedrock vary significantly due to the shape of CZ layers and surface topography. The locations of possible landslides, shown in Fig. 2*Y*, are noted by trapezoidal symbols above the ground surface. Double arrows above the cross-section are orthogonal to the topographic slope.

To better illustrate the varying patterns of seepage flow within CZs, we present the magnitudes and directions of seepage fluxes in two-dimensional cross-sections: an east-west cross-section across the valley and a north–south cross-section along the valley in CZ(stress) in Fig. 4 (yellow and red lines with dots indicating starting locations). The CZ(stress) scenario features significant variations in the thickness of weathered bedrock (Fig. 2*E*). The soil thickness is shallowest at the convex hilltops and locally reaches 65 cm-thick downslope, which is too thin to show seepage fluxes and pressure heads in Fig. 4. In the upper hillslopes bordering the cross-section, the thickness of the weathered bedrock is about 8 m thick, tapering toward tributary channels at their base. The two hills adjacent to the main channel in the east-west cross-section rise about 20 m above the main channel floor. The maximum depth to the fresh bedrock beneath these hills is about 15 m. Only short lengths of either cross-section (indicated by the double arrows above the cross-section) are orthogonal to the topographic slope, underlying weathered bedrock boundary and groundwater table (as shown in the inset map). Hence, the flux vectors record the component of flow aligned to the cross-section, not the maximum fall direction, but comparison with the inset map of groundwater topography suggests that the difference is modest.

The CZ(stress) model predicts the shape of the fresh bedrock boundary, which can differ significantly from the general form of the surface topography (Fig. 4). As a consequence of the great depth to the fresh bedrock boundary, seepage flux follows three-dimensional head gradients that develop in the subsurface rather than aligning with surface topography (see the water table elevation shown as color map of inset map in Fig. 4). The difference between the topographic surface and water table surface is particularly evident under the ridges with deep CZs. For example, in the two lower hills from the east-to-west cross-section (yellow dot transect), the seepage flux from the two upper hillslopes passes under their adjacent channels and contributes to seepage toward the main channel, although its magnitude is smaller than the flux moving from south to north along the larger head gradient (black arrow for the intersection point). Locally, the thinning of weathered bedrock toward the main channel results in more horizontally oriented exfiltrating fluxes at the base of the hillslopes.

The slow seepage fluxes through the underlying unweathered bedrock follow patterns that differ from surface topography, because the fresh bedrock in Fig. 4 is relatively conductive and facilitates groundwater drainage. Beneath the ridges, unsaturated vertical infiltration reaches the deep groundwater fixed by the sloping impermeable bottom flow boundary of the model, 40 m below the local topographic surface. Further downslope, groundwater flow progressively shifts from vertical in the near surface to parallel to the sloping bottom boundary. Fluxes under valley axes are predominantly upward and exfiltrating, resulting from large-scale flow convergence from opposing hillslopes into areas of low total head.

This example highlights the diverse variations in saturation and flux patterns that emerge due to the three-dimensionally varying shapes and structures of deep CZ layers across steep hillslopes, potentially influencing slope stability. As a comparison, CZ(RD4m) in *SI Appendix*, Fig. S9 has thin weathered bedrock, and CZ structures are similar in form to the topography, both the surface and lower boundary condition (Fig. 2*C*). Seepage flux in this case follows a three-dimensional head gradient that aligns with the surface and bottom topographic gradients.

#### Hydrological behavior within the CZ and its impact on seepage flux at the soil–bedrock boundary.

Our modeling results provide insights into the hydrological behavior within the CZ. A detailed explanation is provided in *SI Appendix*, section 3, and a summary is presented here. First, elevated hydraulic head (*h/z* > 0) in the soil mantle is decreased across much of the landscape when there is a deep CZ, even in CZ(RD4m) that includes only up to four meters of weathered bedrock at the water divide. Second, the analysis of seepage vectors and fluxes predicts that seepage fluxes in both soil and bedrock are influenced by deep CZ structures that vary with the distance from ridges or channels. Water seeping out from bedrock to soil across the study areas occurs over relatively small areas in hollows and close to the channels where fresh bedrock is close to the surface. Localized conditions and characteristics of CZ structures and the three-dimensional shapes of topography dictate where exfiltration is most likely to occur.

Last, depending on CZ structure, groundwater in the bedrock seeping into the soil exhibits a wide range of orientations relative to the soil–bedrock interface (*SI Appendix*, Figs. S10 and S11 and Dataset S1). In CZ(soil), normal seepage vectors from the conductive, unweathered bedrock are forced into the soil due to channel boundary conditions, but the seepage vector in the overlying soil is essentially slope parallel (*SI Appendix*, Fig. S10 *B* and *G*). In CZ scenarios with weathered bedrock, the amount of seepage flux in weathered bedrock is generally larger than that in unweathered bedrock but less than the flux within the soil due to the hydraulic conductivity difference. In certain locations, the flux contribution from weathered bedrock influences the head gradients and the orientations of seepage flux developing in the soil above the soil–bedrock boundary (a cell-by-cell comparison between soil above and bedrock below the soil–bedrock interface, provided in *SI Appendix*, Fig. S11). We find that the higher contribution from bedrock seepage to soil flux occurs in some cases, due to the three-dimensional configuration of weathered bedrock in the deep CZ layer (e.g., CZ(stress) in *SI Appendix,* Fig. S10*Y*), causing bedrock seepage to converge and exfiltrate into the soil near channels where weathered bedrock thins.

### Modeled Shallow Landslides.

**Fig. 5.**
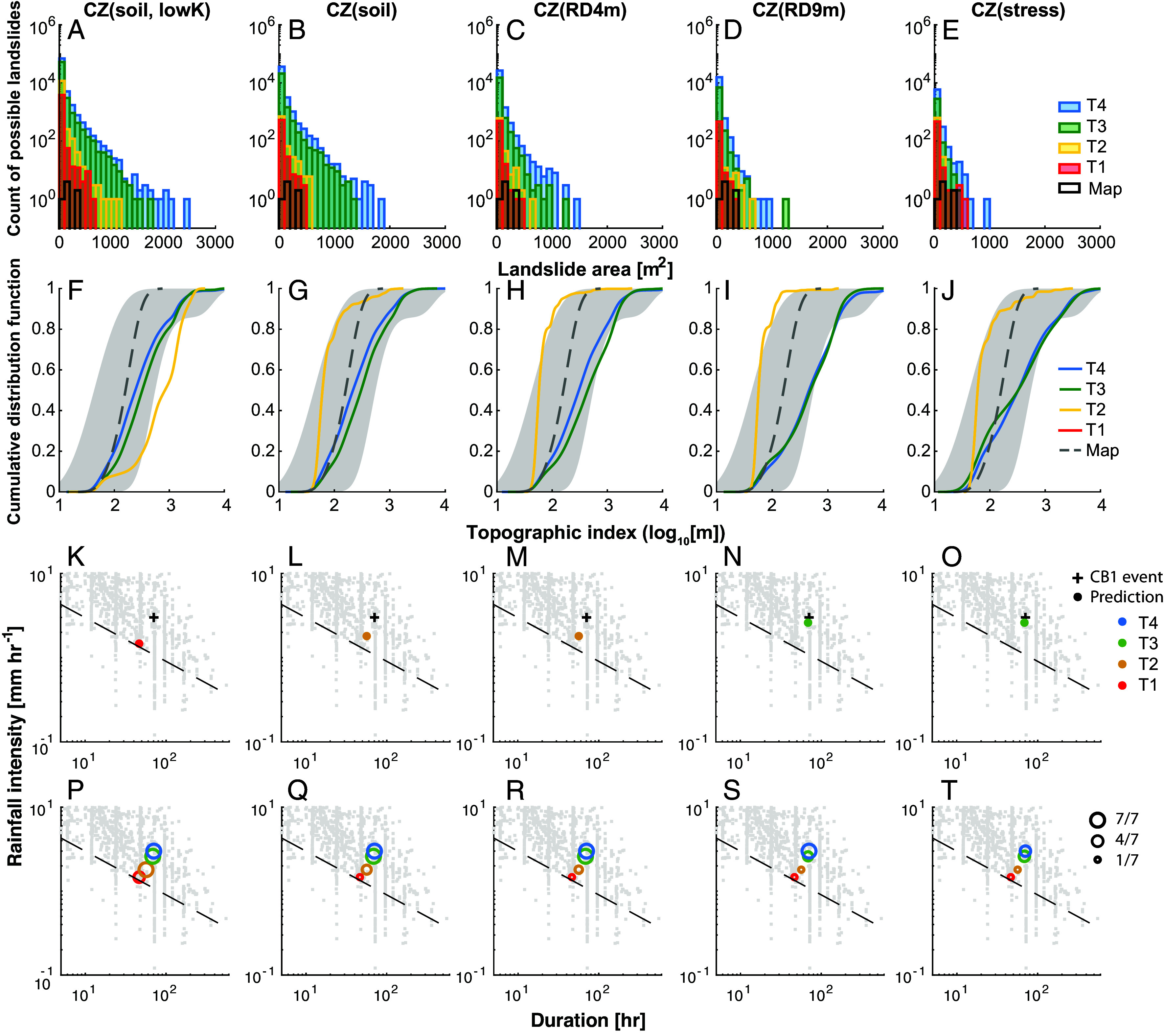
Landslide size distributions, topographic index, and rainfall intensities and durations associated with predicted shallow landslides. (*A*–*E*) Size distributions of all possible discrete landslides, binned by count in 100 m^2^ intervals. (*F*–*J*) Cumulative distribution functions (CDF) of the median topographic index for each landslide cell cluster. (*K*–*T*) Rainfall intensity (*I*) and duration (*D*) values for the CB1 storm and other storms (9). Each column represents a different CZ model scenario. Differently colored bins, lines, and symbols indicate simulation results at different times (T1–T4) during the CB1 storm. Black bins in (*A*–*E*) and gray dashed lines in (*F*–*J*) represent data from seven mapped landslides (Map) at the benchmark site (36). The shaded gray regions in (*F*–*J*) bound the distribution ranges between the 2.5 and 97.5 percentiles. Results at T1 are not shown in (*F*–*J*) due to the relatively small number of predicted landslides at that time. In (*K*–*O*), the black cross indicates the intensity-duration (*ID*) value corresponding to the timing of the actual CB1 landslide (T4), and the colored circle indicates the *ID* value for when each CZ model first predicts the CB1 landslide at the selected intervals of T1 through T4 based on nonzero relative frequency of possible landslides. Circle sizes in (*P*–*T*) represent the number of correctly predicted landslides out of the seven mapped occurrences at the respective times. Gray dots in (*K*–*T*) present a global compilation of *ID* values for shallow landslides and debris flows from Guzzetti (9). The dashed line indicates the *ID* threshold (*I* = 9.9*D*^−0.52^) derived from 35 landslides observed over 10 y in a nearby region (36). Models that include weathered bedrock, predict CB1 landslide timing closer to the actual event. Thinner CZ structures predict more landslides earlier in the storm than deeper CZ structures.

As the CB1 storm progressed from T1 to T4 with increasing rainfall intensity, total unstable areas, the number of possible discrete landslides, and landslide sizes generally increased for all CZ structures (Figs. 2 *U*–*AD* and 5 *A*–*E* and *SI Appendix*, Figs. S6 *U*–*BH* and S12). Early in the CB1 storm, total unstable areas, number of possible predicted discrete landslides, and potential landslide sizes are similar for all CZ structures but diverge considerably later in the storm sequence. This similarity early on is due to predicted landslides from isolated, unconditionally unstable locations due to thick soils on steep hillslopes where local frictional and cohesive resistance was exceeded, even when dry (e.g., the isolated zones in *SI Appendix*, Fig. S6 *U*–*Y*) (12). The predicted landslide size significantly increases for thin CZ structures as the CB1 storm proceeds. Landslide size distributions for thinner CZs have heavy tails, showing the potential for large (>1,000 m^2^) landslides in later times (Fig. 5 *A*–*C*), with increased 95th-percentile value and maximum values (Dataset S2). Heavy-tailed distributions characterize the two endmember CZs lacking a subsurface weathered bedrock. However, CZ structures with thick, weathered bedrock do not exhibit marked increases in landslide size with storm duration (Fig. 5 *D* and *E*), as the areas with flow in the soil (measured by *h/z*) remain limited in extent.

The total unstable area, number of predicted discrete landslides, and potential landslide sizes with thin CZ structures are greater than those from thick CZ structures at all time steps (*SI Appendix*, Figs. S6 *U*–*BH* and S12 and Dataset S2). At the time of the CB1 landslide (T4), the predicted total unstable area as a percentage of the total area based on all possible landslides decreases from 65% in CZ(soil, lowK) to 15% in CZ(stress) with increasing thickness of CZ structures (Fig. 2 *U*–*Y*). Both the total numbers of all possible landslides and pruned landslides decrease with increasing thickness of the CZ (Fig. 2 *U*–*AD* and *SI Appendix*, Figs. S6 *U*–*BH* and S12 *C* and *D*). The pruned landslides, selected by minimum *FOS*, tend to have smaller sizes and are located in areas with a high relative frequency of possible landslides (Fig. 2 *Z*–*AD* and *SI Appendix*, Fig. S6 *AO*–*BH*).

The distribution of predicted landslide locations, using CDF of the median topographic index (an area per unit cell width divided by surface slope) from cells composing each predicted landslide, are shown in Fig. 5 *F*–*J* (percentile ranges reported in Dataset S2). There are differences in CDFs depending on simulation times and CZ structures, although the maximum value of median topographic index is similar. At T2, CDFs from CZs with permeable weathered bedrock [including CZ(soil)] have similar patterns affected by a few predicted landslides with low median topographic indices that are unconditionally unstable (i.e., would fail when dry (12) and *SI Appendix,* Fig. S6 *U–Y*). CZ(soil, lowK) exhibits a higher frequency of predicted landslides and with high median topographic indices compared to the other CZs (yellow line in Fig. 5*F*). The lack of water infiltration into the essentially nonconductive bedrock in CZ (soil, lowK) (*SI Appendix*, Fig. S4 *F* vs. *G*) led to higher flows in the soil and locally slightly higher h/z in the convergent zones, leading to instability (*SI Appendix*, Fig. S6*Z*). At T4, CDFs from all CZ structures show similar cumulative distributions within the 95th percentile range of mapped landslides. CDFs from thick CZ structures tend to have slightly higher median topographic indices than CZs with only soils, due to fewer landslides on upper slopes and in weakly convergent areas.

Fig. 5 *K*–*T* plots storm intensity (*I,* mm/h) and duration (*D,* h) for the four simulated times (T1–T4) of the CB1 storm, alongside a global compilation of events that triggered shallow landslides and debris flows (9). We assume that an unstable area identified by a nonzero relative frequency counts as a success if it appears within a mapped landslide (i.e., Fig. 2 *U*–*Y* and *SI Appendix*, section 2.3). The *ID* (and hence corresponding time event of T1–T4) corresponding to the first successful prediction of unstable area within the CB1 landslide is shown as a filled circle in Fig. 5 *K*–*O*, and the storm period in which it occurred is noted with symbol color. The number of correctly predicted landslides (out of seven) is represented by symbol sizes, with simulated times indicated by colors in Fig. 5 *P*–*T* (*Materials and Methods*).

Thinner CZ structures predict CB1 landslide occurrence earlier in the storms (T1 and T2), while CZ structures with deeper weathered bedrock (e.g., CZ(RD9m) and CZ(Stress)) predict the landslide timing (T3) closer to the actual event (T4, shown with a plus symbol in Fig. 5 *K*–*O*). Thinner CZ structures also predict a greater number of observed landslides earlier in the storm compared to deeper CZ structures (Fig. 5 *P*–*T*). At T4, unstable discrete landslides are predicted for all seven observed landslides in all scenarios, except for CZ(stress), which overlaps with only four landslides. The predicted timing of CB1 landslide and the number of correctly predicted landslides for the given simulation times are the same, whether using nonzero relative frequency or pruned landslides as predictors (*SI Appendix*, Fig. S6 *U*–*BH*).

When comparing total unstable areas and mapped landslides, CZ scenarios with weathered bedrock yield higher accuracy, precision, and F1 scores, but lower recall compared to CZ scenarios without weathered bedrock (Dataset S2 and *SI Appendix*, section 2.3). Six of the mapped landslides failed in storms in previous years in smaller rainstorm events [e.g., another landslide we refer to as CB2 failed twice, during an earlier storm and the event modeled here (31)]. We assume, therefore, that this storm would have caused failure at those sites as well. Model performance (e.g., F1 scores) is similar across CZ scenarios that include weathered bedrock. The CZ model with thick weathered bedrock, CZ(RD9m), predicted the correct location and the closest timing of CB1 landslide in T3 (2 h before T4), as well as all 7 landslides in T4.

## Discussion

### Bedrock Weathering Beneath the Soil Modulates Subsurface Hydrologic Response and Shallow Landslides.

Most slope stability models coupled with hydrologic models predict just the hydrologic responses within the soil from a rainfall event. Models such as TRIGRS primarily focus on transient, unsaturated, vertical infiltration without explicitly considering lateral subsurface flow or permeable bedrock underneath (11). Other models approximate lateral flows within soils under steady-state hydrologic conditions (12) or transient hydrological response during storms (10). These simplified models have been widely applied for regional-scale predictions of locations and timings of shallow landslides due to their computational efficiency (e.g., ref. 44). However, in areas with deep CZs, simplified models may not be sufficient to accurately predict spatial-temporal patterns of shallow landslides and their characteristics. Our work shows that deep CZ structures play critical roles in modulating groundwater storage, influencing groundwater flow paths, and controlling pore-pressure development at the soil–bedrock interface, which affect spatial extents, likely sizes, and timings of shallow landslides.

Our fully integrated three-dimensional transient hydrologic and landslide model highlights the different hydrologic responses and shallow landslide patterns induced by the varying CZ structures. CZs with just soil increase hydrologic connectivity within soils by limiting vertical flow into the deep CZ and forcing water to flow slope-parallel along the soil–bedrock boundary. This process creates wet antecedent conditions and induces the lateral expansion of saturated areas, triggering soil landslides of potentially large sizes, relatively early in a storm (Figs. 2 and 5 and *SI Appendix*, Fig. S6). Conversely, thick CZs with larger groundwater storage capacities may require a longer time to recharge groundwater for exfiltration into soil where weathered bedrock is thin. As a result, thick CZs may limit areas of instability and delay the onset of shallow landslide initiation (Figs. 2 and 5 and *SI Appendix*, Fig. S6). Montgomery et al. (2) documented the CB1 landslide at T4, approximately 1 h after the storm peak and 2 h after peak pressure head in the bedrock. These observations support the influence of exfiltration and downslope groundwater convergence on delayed failure.

Beyond storage-related effects, deep CZs also influence the seasonal evolution of antecedent moisture conditions, which strongly affect landslide timing during storms. In deep conductive CZs, there is a thick, unsaturated, weathered bedrock zone through which incoming rainfall infiltrates to reach the groundwater table. The transmission rate is mediated by the moisture content of the unsaturated zone and has a delayed hydrologic response during storms. Rempe and Dietrich (26) documented that, in a seasonally dry environment, trees exploit this moisture content (this plant-available moisture was termed “rock moisture”), greatly reducing it to a residual moisture level by the end of the dry season. Consequently, during the subsequent wet season, groundwater recharge may be delayed for months as rock moisture increases with successive rainfall events, eventually transmitting significant unsaturated flux to the groundwater.

Incorporating deep CZs and their observed patterns of downslope thinning improves predictions of shallow landslide susceptibility and timings at our sites. Bellugi et al. (38), modeling the same field site, employed model configurations similar to CZ(soil, lowK), with soil atop impermeable bedrock, and a topographically driven, transient hydrologic model (10). They illustrated that small, highly susceptible patches of hillslopes can experience landslides early in storms, prior to peak rainfall. With increasing rainfall intensity (and duration), large landslides develop in hollows and spread up and down hollows and surrounding slopes. These hollows are sites of elevated pore pressures due to subsurface flow convergence and reduced root strength influenced by thickened colluvial soils that accumulate therein (38, 45). Our models with deep CZs reduce the overpredictions of the area of possible discrete landslides (Fig. 2 *U*–*Y*) compared to models with soil only (e.g., total unstable area 23% in CZ(RD9m) vs. 65% in CZ(soil, lowK), Dataset S2). In addition, model scenarios with deep CZs predict the timings of shallow landslide initiations close to the actual event (e.g., 2 h in CZ(RD9m) vs. 24 h in CZ(soil, lowK)).

Locations of high propensity for failure, identified by areas of pruned landslides and high relative frequency of possible landslides (Fig. 2 *Z*–*AD*), are similarly found mostly in convergent areas across the CZ scenarios. Convergent areas in hollows typically have thicker soils and high flow accumulation both in soil and weathered bedrock (e.g., refs. 45 and 46). This implies that the spatial similarity of high landslide propensity is likely due to the combined effects of subsurface flow driven by downslope thinning of CZs, topographic gradient, and soil thickness and parameterization rather than soil thickness variations alone.

### Limitations, Implications, and Future Directions for Improved Landslide Prediction Models.

Our fully coupled models integrating CZ structure, three-dimensional transient hydrology, and multidimensional slope stability capture the first-order control exerted by spatially varying weathered bedrock thickness on the location, size, and timing of shallow landslides. However, these models cannot fully account for certain processes in natural landscapes due to limited data and simplifying assumptions. There are remaining challenges of overprediction of possible landslides, the lack of consideration for preferential flow within discrete fracture networks, uncertainties in model parameterization, and the limited comparison between modeled simulations and mapped landslides from various prior storms (*SI Appendix,* section 4).

We acknowledge that several of our model parameters are assigned in a simplified manner and are subject to uncertainty, including soil cohesion, friction angle, and hydraulic conductivity parameterizations for the CZ layers (*SI Appendix*, sections 2 and 4). In our model, we assigned distinct saturated hydraulic conductivities and van Genuchten soil water retention curves to each CZ layer. However, we assumed no vertical gradient in hydraulic conductivity within individual layers, which contrasts with the vertical variability inferred from slug tests (1, 31). Previous studies at CB1 (e.g., refs. 28 and 31) have noted that hydraulic conductivity measurements for saprolite and weathered bedrock are too scattered to define a single representative value. Reported values span over five orders of magnitude, likely reflecting the influence of fracture flow, material heterogeneity, weathering transitions, and variable saturation conditions. The saturated hydraulic conductivities assigned to bedrock were measured near the surface and thus likely represent the upper end of the true range (*SI Appendix*, section 2). Lower conductivities in both fresh and weathered bedrock at depth would increase subsurface flow within the soil and reduce the influence of the deeper flux vectors, including potential exfiltration from weathered bedrock.

In addition, although shallow landslide activity at the soil–bedrock boundary is limited for the case of deep CZs, bedrock weathering and groundwater dynamics in the deep CZ may influence the rates and movement of deep-seated landslides. We did not model the behaviors of deep-seated landslides. However, previous studies have shown that bedrock fractures and weathering influence the mechanical strengths of rock mass and bedrock landslide thickness (47, 48), and that water table rise and pore-pressure developments in deep CZs are important in modulating the rate and timing of deep-seated, slow-moving landslides (49, 50).

Our work shows that CZ structure can lead to distinct differences in the location, size, and timing of predicted shallow landslides, suggesting that physical insights into CZs may be inferred from shallow landslide observations. First, mapping the spatial occurrence of shallow landslides after a storm event may help infer the presence of a deep CZ control. If the CZ thins downslope, as is often proposed in steep landscapes, hillslopes bordering channels are likely underlain by fresh bedrock. If a deep CZ is absent, landslides are favored in areas of topographic convergence (e.g., ref. 41), but landslides may also occur outside of hollows and toward divides. Conversely, if shallow landslides are rare to absent outside of hollows but occur near the base of hillslopes, this suggests a CZ that may deepen toward the ridge. Second, variations in CZ depth may partly explain the observed variability in rainfall intensity–duration and cumulative rainfall thresholds for triggering shallow landslides. These thresholds are commonly used in forecasting tools for shallow landslide and debris flow initiation (7, 8). However, global compilations show substantial variability in threshold values (6, 9). Differences in CZ structure—such as shallow versus deep CZs—may influence whether shallow landslides initiate at lower or higher rainfall intensities for a given duration or occur earlier or later during a storm.

This deep CZ perspective on subsurface groundwater flow and shallow landslides is often overlooked when assessing landslide susceptibility and predicting hazard magnitude and timing. Current landslide susceptibility models that do not account for the deep CZ may represent higher-end susceptibility estimates. Although it is challenging to constrain variations in deep CZ thickness, several studies have documented regional or hillslope-scale variations in the thickness of permeable, weathered bedrock (e.g., where the interface between weathered and unweathered bedrock is subdued, inverted, or unrelated to surface topography) (4, 16–18, 24, 25, 33). These studies highlight key factors, including lithology, climate, tectonics, hydrology, and topography, that control spatial variations in the deep CZ. With the growing availability of remote-sensing data, field sensor deployments, and enhanced computational capabilities, future campaigns can integrate multiple datasets. Resulting new datasets include carefully mapped landslides using high-resolution topography, detailed soil depth and root strength measurements, accurate landslide timing, precipitation or weather station data, and comprehensive assessments of CZ structures. When combined with the coupled process modeling used in this study, these datasets may significantly improve landslide susceptibility assessments and early warning systems in specific regions.

## Materials and Methods

We generated five theoretical CZ structures using different assumptions of underlying bedrock properties and structures. Both CZ(soil) and CZ(soil, lowK) consist of soil over unweathered bedrock, but with different saturated conductivity (5.0 × 10^−7^ m/s vs. 5.0 × 10^−12^ m/s) (28, 30). In three CZ structures, soil overlies weathered bedrock, for which the saturated hydraulic conductivity is 7.2 × 10^−5^ m/s (*SI Appendix*, Table S2). Rempe and Dietrich (24) proposed a weathering model based on bedrock drainage and channel incision and calculated the relief of the weathered–unweathered bedrock boundary. We assume a constant slope profile extending from zero weathered bedrock thickness at the base of the hillslope to either the 9 m-deep for CZ(RD9m) or 4 m-deep for CZ(RD4m) at the CB1 borehole (35). For CZ(stress), the weathered bedrock thickness is based on topographic stress using three-dimensional subsurface stress field modeling, calibrated based on regional-scale in-situ stress data (16, 51). The descriptions and configurations of CZ model scenarios explored in this study are presented in *SI Appendix*, section 2.1 and Table S3. We use the hydrologic model GEOtop 2.0 (39) to simulate variably saturated water fluxes through CZ structures and predict the hydrologic variables at specific times of interest (details in *SI Appendix*, section 2.2). We modify a multidimensional slope stability model, coupled with a spectral search algorithm, to incorporate spatially variable seepage forces from our hydrologic model and predict the stability of clusters of adjacent grid cells (37, 38, 41, 42) (*SI Appendix*, section 2.3).

There were a total seven mapped landslides at the benchmark site between 1987 and 1996 (36). Both CB1 and CB2 experienced debris flow events during the CB1 storm in 1996. But, CB2 had failed previously in February 1992 (31). Six landslides that occurred earlier than the CB1 storms were driven by different rainstorm conditions and may not be directly comparable to our simulation due to differing antecedent conditions, rainfall intensities, and root strengths (discussion in the *SI Appendix*, section 4). Montgomery et al. (36) showed that the frequency of landslides during this period increased following commercial forest harvesting. Because these landslides occurred with rainfall intensities at or below those of the CB1 storms (36), they could have occurred during the CB1 storms if these slope failures had not already occurred. We assume these landslides provide information on landslide sizes and locations for plausible landslides that can be compared with our model predictions. We assess the model’s performance for landslide prediction by comparing the predicted total unstable areas, based on the nonzero relative frequency of landslides, with the seven observed landslides. The predicted timing of the CB1 landslide and the number of correctly predicted landslides for the given simulation times are the same, regardless of whether nonzero relative frequency or pruned landslides are used. The details of the assessment, including performance metrics such as accuracy, precision, recall, and F1 scores, are described in the *SI Appendix*, section 2.3.

## Supplementary Material

Appendix 01 (PDF)

Dataset S01 (XLSX)

Dataset S02 (XLSX)

## Data Availability

Previously published data (1, 2, 27, 28) and models (16, 24, 39, 41) were used for this work. All data and model information are included in the article and/or *SI Appendix*.
