# Supplementary material for: Deep critical zone controls on shallow landslides
Source: Proc Natl Acad Sci U S A. 2026 Mar 18;123(12):e2524542123. doi: 10.1073/pnas.2524542123 (PMC13012049; doi:10.1073/pnas.2524542123)
Supplement: Supplementary file 1 — Appendix 01 (PDF) [file pnas.2524542123.sapp.pdf]

Supporting Information for

Deep critical zone controls on shallow landslides

Seulgi Moon[1*], Giuseppe Formetta[2*], Justin T. Higa[1], Riccardo Busti[2], Dino G. Bellugi[3], David G. Milledge[4], Brian A. Ebel[5], William E. Dietrich[3,6]

[1]Department of Earth, Planetary, and Space Science, University of California, Los Angeles, Los Angeles, CA 90095, USA.

[2]Department of Civil, Environmental and Mechanical Engineering, University of Trento, Via Mesiano 77, I-38123 Trento, Italy.

[3]Department of Geography, University of California, Berkeley, CA 94720, USA.

[4]School of Engineering, Newcastle University, Newcastle upon Tyne, NE1 7RU, United Kingdom.

[5]United States Geological Survey, Water Resources Mission Area, Burlington, VT 05405, USA.

[6]Department of Earth and Planetary Science, University of California, Berkeley, CA 94720, USA.

Co-corresponding authors: *Seulgi Moon (sgmoon@g.ucla.edu), *Giuseppe Formetta (giuseppe.formetta@unitn.it), * William E. Dietrich (bill@eps.berkeley.edu)

This PDF file includes:

Supporting text

Figures S1 to S12

Tables S1 to S4

SI References

Supporting Text

1. Study area

The Oregon Coast Range is a highly dissected, steep, forested, soil-mantled mountain range that is prone to deep-seated and shallow soil landslides (e.g., *1*). Here, we examine a ~0.04 km^2 catchment, ~15 km NE of Coos Bay, Oregon, USA (where annual rainfall is about 1600 mm), as a benchmark site for modeling shallow landslides (Figs. 1A, S1A). We chose this site because of its extensive history as a research area for weathering, hydrologic, and landslide studies (e.g., *2, 3-13*). Specifically, a zero-order catchment known as CB1 has been extensively studied through numerous field campaigns. This site is underlain by rhythmically bedded graywacke sandstone of the Eocene Tyee Formation (*14*) with an average hillslope gradient of ~43°. Local exposures near CB1 indicate sandstone beds with a dip of 8°- 15° into the slope (*3*). Montgomery et al. (*15*) reported 195 hand-augered soil thickness measurements near CB1. Soil is thin (< 0.5 m) on adjacent spur ridges and thick (> 1.0 m) in the CB1 hollow. Soil is produced by tree throw and mountain beaver (*Aplodontia rufa*) burrowing (*7*), which leads to a nonlinear increase in transport (*6*) on steepening slopes as well as thickened soils in the topographically convergent hollows.

Field observations and modeling studies at CB1 document the contribution of root strength to slope stability (*16-18*). CB1 and the entire benchmark area shown in Fig. 1A was clear-cut harvested in 1987 and replanted in 1988. Montgomery et al. (*5*) mapped 35 shallow landslides from 1987 to 1996 in the Mettman Ridge area around the benchmark site (Fig. 1A). They proposed that a significant reduction in root cohesion due to recent timber harvest led to the mapped landslides.

Previous fieldwork studies also demonstrated the involvement of groundwater in shallow landslide activity in CB1 at the benchmark site (*8, 9, 15*). On 20 February 1992, a landslide occurred at CB2, a catchment ~100 m northwest of CB1 (Fig. 1B) (*8*). The peak hourly rainfall intensity at the time of the CB2 landslide was 15.5 mm/hr, and the intensity-duration characteristic of the storm was ~3.5 mm/hr for 9 to 16 hours (*8*). Inspection soon after failure revealed water exfiltrating from bedrock fractures in the landslide scar (*9*). A record storm from 15 to 20 November 1996 (Fig. 1C) caused widespread landsliding across the Oregon Coast Range, including landslides at CB1 and CB2. The final dimensions of the CB1 landslide were 5 – 9 m-wide and 21 – 23 m-long with an area of 157 m^2, but field analysis suggested a smaller 58 m^2 initial scar in the upper portion of the mapped landslide (Fig. 1B) (*9*). Groundwater exfiltration was also observed in the CB1 failure scar, which corresponds to a previously monitored area of local elevated groundwater in the soil (*15*).

Direct field monitoring at CB1 indicated that exfiltration from fracture flow in the underlying weathered bedrock contributed to the soil landslide. Piezometers installed in the CB1 hollow had measured pressure heads in soil nearly continuously from 1990 – 1996, until this landslide destroyed most of the field infrastructure (*8, 9, 15*). The positive pressure head at the soil-bedrock boundary (h [m]) in a soil thickness (z [m]), hereafter h/z, was highest above a zone of

fractured bedrock later exposed by the landslide (*h/z* = 0.4 – 0.75) (*9*). The lowest *h/z* was located ~5 m away (0.1 – 0.2), where fewer bedrock fractures were documented. Such patchy saturation agrees with fracture exfiltration at CB1 that occurred during sprinkler experiments (*8, 10, 15, 19*) and hydrologic modeling in soil and bedrock (*11, 20, 21*). These instrumental records indicate that the CB1 landslide occurred approximately one hour after the storm peak and two hours after peak pressure head in the bedrock (*9*). Anderson et al. (*2*) used tracer studies during sprinkler experiments and natural rainstorms and proposed that along the hollow axis, groundwater would locally exfiltrate into the soil, re-infiltrate into bedrock a short distance downslope, and then exfiltrate again farther downslope to generate patchy partial saturation (*h/z* <1) in soil. These findings suggest that exfiltration from underlying bedrock is a likely driver of shallow landsliding.

Such detailed documentation of soil parameters, saturation, and landslide timing allows researchers to model soil slope stability at the benchmark site and CB1. Several studies have used one-dimensional, infinite-slope limit equilibrium models with spatially variable saturated flows (*4, 22-24*) and two-dimensional continuum deformation (*25*) at this site. Montgomery et al. (*9*) used a three-dimensional model that included lateral root strength but not lateral soil frictional strength and measured *h/z* to predict the location of the shallow landslide. Recent work developed a three-dimensional limit equilibrium model that accounts for resisting forces on all edges of a discrete landslide to predict the CB1 landslide using measured saturation, soil thickness, and root strength (*26*). This model is coupled to a spectral search algorithm as a computationally efficient method to identify mutually exclusive or overlapping clusters of unstable soil cells (*27, 28*). Such an efficient approach enabled three-dimensional landslide modeling driven by individual rainstorm events across the benchmark site and has been used to evaluate controls on landslide size and location by factors including rainfall intensity, soil thickness, soil strength, and root cohesion (*16, 27, 28*). However, these modeling efforts assumed all runoff was confined to the soil mantle, which is a useful simplification for modeling purposes. This configuration is inconsistent with extensive field monitoring, which showed essentially all runoff traveling through the weathered bedrock (*2, 15*).

Previous studies documented spatial variation of the CZ structure at CB1, including colluvium, saprolite, and weathered and fractured bedrock thickness (*2, 3, 15*). Montgomery et al. (*15*) installed 195 piezometers distributed among 86 nests in the soil mantle and 28 piezometers into bedrock, with one 35 m-deep well at the hillslope divide. The drilling results showed that weathered bedrock thins downslope at CB1. At the drainage divide at the top of CB1, the weathered bedrock transitions from pervasively oxidized to fractured and partially oxidized sandstone with depth (*3*). At this CB1 borehole, the boundary between pervasively oxidized and fractured bedrock is 4.5 m-deep, and that between open fractured and unweathered bedrock is 9 m-deep. Porosity observations from this borehole and nearby soil pits agree with slug tests that suggest hydraulic conductivity increases towards the surface. However, hydraulic conductivities measured from the same weathered layer can vary by several orders of magnitude (*2, 8, 11, 15, 20*). Tracer studies injected into groundwater in upslope weathered bedrock documented water exfiltrating at spring locations into the soil mantle via open fractures in the bedrock (*2*). The peak velocity through fractured bedrock was estimated to be ~1 x 10^{-3} m/s based on two bromide

injection experiments (*29*), which are orders of magnitude higher than saturated unweathered bedrock ($5x10^{-7}$ m/s) and even higher than the averaged soil conductivity (3.4×10^{-4} m/s).

Rempe and Dietrich (*30*) proposed an analytical model that predicts the fresh bedrock boundary in our study area. During landscape uplift, channel incision into saturated fresh bedrock induces lateral head gradients that slowly drain the fresh bedrock, allowing oxygen and meteoric water to infiltrate and drive chemical and mechanical weathering of the bedrock. Such weathering creates porosity, causing the bedrock to hold and transmit precipitation-driven groundwater flows. Extensive fieldwork in the Coos Bay area enabled them to parameterize that model, but the ratio of saturated conductivity to bedrock porosity of the fresh bedrock had to be calibrated. They predicted a weathered bedrock layer 9 m-thick at the ridgeline that thins downslope towards the hollow, similar to field observations.

2. Details of the models

2.1. Critical zone structures and properties

We generated five theoretical CZ structures using different assumptions of underlying bedrock properties and structures. CZ(soil) consists of soil over unweathered bedrock with a saturated conductivity of ($5.0x10^{-7}$ m/s), similar to that used in previous hydrologic models at this site based on field measurements (*11, 20*). The hydraulic conductivity of $5.0x10^{-7}$ m/s is the upper side of the reported range for the sandstone (*31*) and likely higher than expected for fresh, unweathered sandstone bedrock at our site (*8, 30*). In CZ(soil, lowK), saturated conductivity was $5.0x10^{-12}$ m/s (more typical of shale-dominated bedrock (*31*)). Three CZ structures consist of soil over weathered bedrock and unweathered bedrock with saturated hydraulic conductivities of $7.2x10^{-5}$ m/s and $5.0x10^{-7}$ m/s, respectively (Table S2). Rempe and Dietrich (*30*) proposed a weathering model based on bedrock drainage and channel incision and calculated the relief of the weathered-unweathered bedrock boundary, Z_b [m]. For CZ(RD9m), weathered bedrock thickness is based on a constant Z_b/Z_s of 0.8 (i.e., the relief of the unweathered bedrock surface, Z_b, is 80% of the topographic relief of the ground surface, Z_s), which is similar to 0.83 from Rempe and Dietrich (*30*). CZ(RD4m) is similar to CZ(RD9m) but with Z_b/Z_s of 0.9. We assume a constant slope profile extending from zero at the base of the hillslope (i.e., no weathered bedrock) to either the 9 m-deep for CZ(RD9m) or 4 m-deep for CZ(RD4m) at the CB1 borehole (which is similar to the depth to fractured and pervasively weathered layers reported by Anderson et al. (*3*)). Hillslopes are defined as being bordered by a channel. Using these ratios, we approximate the thickness variations of the benchmark catchment from channel to hilltop. For CZ(stress), weathered bedrock thickness is based on topographic stress using three-dimensional subsurface stress field modeling calibrated for the benchmark site, following St. Clair et al. (*32*) and *in-situ* stress measurements from hydrofracturing and overcoring available on a regional scale (*33-35*). Below, we provide detailed information and procedures for calibrating the critical zone model for this site.

2.1.1 Soil

We model the spatial distribution of soil thickness following Dietrich et al. (*36*) and Bellugi et al. (*28*) using a landscape evolution model that couples an exponential soil production function (*7*) and a nonlinear soil diffusion model (*6, 36*). Following Bellugi et al. (*27, 28*) and citations therein, the rate of soil production that decays exponentially with depth is calculated as

$$-\frac{\partial z_b}{\partial t} = \varepsilon e^{-\alpha z cos(\theta)}, \qquad \text{(Eq. S1)}$$

where the height of the soil-bedrock boundary (bedrock can be unweathered (fresh) or weathered) above a datum is z_b [m], time is t [yr], vertical soil thickness is z [m], soil production rate at 0 m soil thickness is ε [m/yr] (0.268 mm/yr), the soil production rate constant is α [1/m] (0.0003/m) (*7*) and topographic slope is θ [deg.]. Annual nonlinear soil flux per unit contour width, q [m^2/yr], is calculated as

$$q = \frac{D\nabla z_t}{1-(|\nabla z_t|/S_c)^2}, \qquad \text{(Eq. S2)}$$

where the diffusion coefficient is D [m^2/yr] (0.0032 m^2/yr), topographic elevation above a datum is z_t [m], and the critical slope is S_c [m/m] (1.25 m/m) (*6*).

This coupled model simulates soil production at every cell based on its soil depth, transports this soil based on topographic slope, and outputs a map of predicted soil thickness. We model soil production and transport for 6000 years to match field measurements of soil depth at this site (*15*). We use a post-landslide, 2 m-resolution LiDAR digital elevation model (DEM) of the benchmark site (*6*) and parameters calibrated from measurements of local erosion rates (0.05 – 0.15 mm/yr) (*6, 7, 37*). Channels act as boundary conditions for hillslope transport. Soils arriving at channels are removed, leaving a minimum soil thickness of 0.02 m. To extract channels, we initially calculate drainage pathways (*38*) and then manually match the locations of channel heads based on field-mapped locations (*5*).

2.1.2 Weathered bedrock model: bedrock drainage

We use a weathering model based on bedrock drainage and channel incision, following Rempe and Dietrich (*30*), to include weathered bedrock in our landslide modeling. This model uses a one-dimensional, steady-state form of the Boussinesq equation for groundwater flow to solve for the elevation of the groundwater table (*39*). We assume that water below this boundary is chemically equilibrated with the surrounding, saturated bedrock and thus unreactive. Therefore, the calculated groundwater table is a proxy for the weathered-unweathered bedrock boundary.

Rempe and Dietrich (*30*) calibrated this weathering model based on field data at CB1, including measurements of channel incision rate, rock and soil bulk density (*3*), soil diffusivity, critical slope (*6*), and hillslope length. From these parameters, they estimate a ratio of hydraulic conductivity to porosity, K/θ [m/s], to measure the ratio of subsurface flow capacity to storage

capacity. Subsequently, they calculate the relief of the unweathered bedrock surface, Z_b [m], and topographic relief of the ground surface, Z_s [m], along a two-dimensional section starting from the CB1 borehole and ending at the channel head of the CB1 hollow. The ratio of Z_b/Z_s at the CB1 ridgeline was estimated to be 0.83 (*30*). We expand this approach to estimate the three-dimensional CZ structure across the benchmark site.

We follow Rempe and Dietrich (*30*) to create two CZ structures based on a calibration with different weathered boundaries in the CB1 borehole. To do this, we first calculate channel networks in a LiDAR DEM (with a larger extent than the benchmark site to capture surrounding streams) using a drainage area incision threshold of 1500 m^2 using the MATLAB TopoToolbox (*40*). This threshold places the head of the CB1 channel near the bottom of the mapped landslide, similar to mapped channels (*5*). Then, we calculate a map of Z_s as the topographic relief of any grid cell and the channel cell into which water from that particular cell will flow. We explore a simple case to calculate Z_b from Z_s by assuming that constant ratios of Z_b/Z_s at the CB1 borehole are equivalent to Z_b/Z_s across the benchmark site. We assume a constant slope from the elevation of the ridgeline Z_b to the channel at the base of the hillslope, leading to a wedge-shaped thinning of the weathered bedrock to zero at the channel.

We assume two rounded values for Z_b/Z_s at the CB1 borehole, 0.9 and 0.8, to construct our CZ structures. These ratios are calibrated to match the 4.5 m-deep boundary between pervasively oxidized and fractured bedrock and the 9 m-deep boundary between fractured and unweathered bedrock in the CB1 borehole (*3*). The 9 m-deep boundary is equivalent to the depth of unweathered bedrock modeled by Rempe and Dietrich (*30*). Anderson et al. (*3*) noted that fracture flow in bedrock is a dominant hydrologic pathway, and the hydraulic conductivity in the saprolite and pervasively oxidized and fractured bedrock layer is likely much higher than that in unweathered bedrock.

We multiply the map of Z_s by our Z_b/Z_s ratios to obtain two maps of estimated Z_b. Lastly, we subtract Z_b from Z_s to calculate the modeled thickness of weathered bedrock and smooth this map using an unweighted mean of a moving circular window with a radius of 5 m. We output two maps of depth to unweathered bedrock for estimating CZ architectures with weathered bedrock across the benchmark. The CZ(RD9m) and CZ(RD4m) structures, as applied here, are defined by distance from an adjacent channel (Fig. 2C, D). Hollows are topographic convergent zones that are likely cut by periodic debris flows. Hence, they record episodic channel network extension events (*41, 42*). For simplicity, we did not consider the temporal frequency or spatial distributions of these periodic events when constructing CZ profiles.

This simplified approach to the bedrock drainage model assumes a constant Z_b/Z_s ratio for every ridgeline, ignoring variations of Z_b/Z_s along the hillslope length. We examine the distribution of hillslope length throughout the benchmark site. We find that the mode of hillslope length is 71 m, similar to that reported by Rempe and Dietrich (*30*) as 76 m (Fig. S1). We refer readers to Rempe and Dietrich (*30*), who provide a full explanation of the approach.

2.1.3 Weathered bedrock model: topographic stress

We model the variation of weathered bedrock thickness based on three-dimensional subsurface stress field calculations following the approach described in St. Clair et al. (*32*) and Moon et al. (*43*). These studies examine the topographic stress field, calculated as the combination of ambient tectonic compression, gravitational loading, and topographic perturbations on geologic stress fields assuming simple material properties (e.g., linear elastic and homogenous rock). Several studies have shown that topographic stress can vary depending on location and likely influences the subsurface extent of open fractures (*32, 43, 44*). Comparisons between topographic stress predictions and near-surface seismic refraction tomography at the benchmark site show the potential for bedrock weathering to be influenced by the least compressive principal stresses (LCS) from the topographic stress field (*45*). However, the correspondence is not as strong as in other studies (*32*). Nonetheless, we use topographic stress as a weathering mechanism to construct an endmember CZ structure with deep and variable weathered bedrock.

We calibrate a topographic stress model for the benchmark site. To do this, we first estimate ambient tectonic stresses and depth gradients using *in-situ* stress measurements from hydrofracturing or overcoring across the west coast of the United States and Canada. We compile 14 *in-situ* stress measurements from the World Stress Map 2016 (*33*) and previous work (*34, 35*). Then, we use a linear least squares regression to determine a best-fit tectonic stress magnitude and depth gradient for maximum horizontal, minimum horizontal, and vertical stresses (Fig. S2). This procedure provides the ambient tectonic stress for topographic stress.

Next, we transform the 2 m-resolution LiDAR DEM to model the topographic stress field with the ambient tectonic conditions above following Moon et al. (*43*). We convert the DEM into a triangular mesh with leg lengths of ~5 m that increase to ~50 m outside the benchmark to reduce computational time. Then, we calculate topographic stresses within an observation grid encompassing the benchmark site. This grid has a horizontal resolution of 5 m x 5 m and a vertical resolution of 3 m (64 elements in the E-W direction, 64 elements N-S, and 99 elements vertically, extending ~100 m beneath the lowest point in the benchmark site).

Lastly, we simulate the topographic stress field under the benchmark site, assuming linear-elastic, homogenous, and isotropic material using the boundary element model Poly3D (*46*). We used inputs of the constrained ambient tectonic conditions, topographic mesh, and observation grids to obtain a total stress field. From these total stresses, we index the LCS magnitude 9 m-deep in the CB1 borehole and use this LCS value of 0.11 MPa as a threshold for fracture openness, assuming LCS represents bedrock fracturing across the entire site (where positive stress values indicate compression). Thus, we create an isosurface where LCS equals this threshold value as a spatially variable map of depth to unweathered bedrock. We mask out this depth to unweathered bedrock within 5 m of the surface to avoid artifacts induced by the topographic mesh by setting areas < 5 m-deep to a shallow value of 0.02 m. Then, we resample the maps of depth to unweathered bedrock from 5 m- to 2 m-resolution and smooth the maps using a moving circular window of radius 5 m. Finally, we output a map of depth to unweathered bedrock across the benchmark based on topographic stress.

2.2. Hydrologic model, parameterization, rainfall conditions, and output

We use the hydrologic model GEOtop 2.0 (*47*) to simulate variably saturated water fluxes through CZ structures. This model uses a three-dimensional finite volume approach to predict surface and subsurface flow, accounting for the lag time between overland and subsurface flow coupling (*48*). GEOtop 2.0 calculates unsaturated and saturated flow by solving the three-dimensional Richards equations (*49*), using the van Genuchten soil water retention curves (SWRC, (*50*)) and the Mualem soil water conductivity function (SWCF, (*51*)). The model also calculates overland flow by extending the validity of Darcy's Law to surface flow and channel routing using the shallow water equation, neglecting inertia (*47*). GEOtop 2.0 has been utilized to calculate soil pressure head at multiple layers, the infinite-slope factor of safety, and landslide occurrences (*52*). GEOtop 2.0 also produces results in good agreement with a suite of other three-dimensional hydrologic models (*53*). Below is detailed information on our model setup, parameterization, and input data.

First, we divide the site into 9,696 CZ column types with specific soil SWRC and SWCF that describe the soil and weathered and unweathered bedrock column at every cell in the model area with a horizontal resolution of 2 m. We discretize the CZ column into cells 40 m-deep, where the vertical resolution of cells increases with depth from 0.05 m to 0.5 m to decrease computational time (Table S1). We calculate saturated and unsaturated subsurface flow through layered CZs with water content and hydraulic conductivity that varies with matric potential (*47*).

Second, we impose impermeable boundary conditions at all borders except for the outlet where water can flow through the channel, consistent with the approach of Ebel et al., (*20*). The bottom boundary is set at a depth of 40 m from the surface, which means it has the same topography as the ground surface, while the side boundary follows the catchment boundary (divide) of the entire model domain. Hence, the bottom boundary is a complex surface that slopes beneath the ridge to the channel, influencing the groundwater flux patterns within the bedrock near the bottom boundary. The choice of a 40-m depth is guided by local field observations from the 35-m CB1 core described in Anderson et al. (*3*). The water table is located approximately 20 m below the surface at the catchment divide (*8*). Core samples collected below 24 m show an absence of oxidation staining in bedrock; those collected between 30 and 35 m show cemented fractures and the presence of pyrite, indicating minimal chemical weathering (*2, 3*). This pattern suggests limited water flow at these depths. In addition, Montgomery et al. (*15*) quantified runoff coefficients from upper and lower weirs at CB1 and from a weir at CB2 during natural and experimental rainfall events. Their results indicate that the combined contributions of evapotranspiration and deep groundwater leakage account for less than ~20% of incident rainfall. Based on this water balance, we assume that an impermeable lateral boundary and a 40-m bottom boundary are reasonable for the model domain. At the channel outlet, we designate the top 24 m as a free-flux boundary condition and bottom 16 m as a no-flux boundary condition. This choice aligns with the 20–24 m depth at CB1, where groundwater and the absence of oxidation indicate limited flow beneath this level (*3, 8, 29*), although we acknowledge that the outlet is located downstream from the CB1 borehole. These boundary conditions are based on available local observations at CB1, which may differ at other sites.

Third, we impose realistic hydrologic parameters for residual and saturated water content, field capacity, soil water retention curves (SWRC), specific storativity, and field-averaged porosity and hydraulic conductivity to each geologic layer in a CZ column type following previous studies (Table S2) (*3, 10, 11, 15, 20*). The van Genuchten model parameterization for SWRC is expressed as:

$$\frac{\theta-\theta_R}{\theta_S-\theta_R} = [1 + (\alpha|h|)^n]^{-m}, m = 1 - 1/n \quad \text{(Eq. S3)}$$

where h [L] is the pressure head, θ [−] is the water content, θ_R [−] and θ_S [−] are the residual and saturated water content, α [1/L] is the scaling parameter related to the inverse of the air-entry pressure, n [−] is a dimensionless measure of the pore-size distribution.

We adopt the values presented by Ebel et al. (*20*), which used the hydrologic properties for the soil based on compiled field measurements at the CB1 sites (*3, 10, 11, 15*) and those for weathered and unweathered rocks based on field measurements from another site (i.e., Yucca Mountain, Nevada, USA) (*54*). Direct measurements of van Genuchten parameters for weathered or fresh bedrock at CB1 are not available, and such values are notoriously difficult to obtain. We chose to use a value from shallower, more conductive saprolite to represent our weathered bedrock (7.2 x 10^{-5} m/s). The assigned value of 5 x 10^{-7} m/s for fresh bedrock is based on the observed decline rate of the water table in the deep well during the dry season. This value is probably relatively high considering the observed minimum value of 9.4 x 10^{-9} m/s for unweathered bedrock (8).

Lastly, we use the natural rainfall record of the CB1 storm from 00:00 13 September 1996 to 20:00 18 November 1996, recorded at ten-minute intervals (Fig. 1C). We follow a two-month initial warm-up period, as shown in the previous study (*20*) using a 10-minute model time step. Ebel et al. (*20*) demonstrated that a three-dimensional infiltration model solving the same governing equations as GEOtop accurately reproduced pressure heads and soil moisture at the CB1 monitoring sites at the start of the controlled sprinkler experiments, using a two-month spin-up period. The rainfall record was collected using tipping bucket rain gauges at CB1 (*9, 15*). The instrument recorded a light antecedent rainfall beginning on 12 November, followed by the onset of the continuous rainstorm around 21:30 on 15 November 1996, which then accumulated until the CB1 landslide swept away the instrument at 20:00 on 18 November 1996. Between 16 to 18 November 1996, the rain gauge measured a total of 225 mm of rainfall, with a maximum 24 hr intensity of 6.0 mm/hr (*9*). The record indicates that the CB1 rainstorm intensity increased as the storm progressed over time (Fig. 1C).

Based on the inputs of CZ column types, hydrologic parameters, and rainfall records, we use GEOtop 2.0 to simulate the hydrologic response and calculate pore-water pressure and soil water content within our CZ structures. We use the simulation outputs at specific times of interest for landslide modeling (see next section). We use the model outputs at four different times: 17 November 1996 20:00 and 18 November 1996 06:00, 18:00, and 20:00 (hereafter, T1, T2, T3, T4; Fig. 1C). These times represent 24, 14, 2, and 0 hr before the CB1 landslide and 46.5, 56.5,

68.5, and 70.5 hours after the onset of a continuous, significant rainstorm (T0, 21:30 15 November 1996, Fig. 1C). They have corresponding rainfall intensities of 1.47, 1.80, 2.60, and 3.00 mm/hr, considering their rainfall duration after T0. According to a 10-year historical inventory of 35 landslides mapped by Montgomery et al. (*5*), the intensity (*I,* mm/hr) and duration (*D,* hours) of rainfall events that triggered shallow landslides in this area are estimated as $I = 9.9D^{-0.52}$. The rainfall intensities for T1 – T4 averaged over the duration since T0 are 9, 48, 137, and 177% higher than these estimated landslide-triggering rainfall intensity thresholds. Montgomery et al. (*5*) found that the most intense 24-h storms associated with landslides occurred over six years and reported that 24-h averaged rainfall intensities greater than 2.37 mm/hr likely produced these landslides. As a reference, the 24 hr-averaged rainfall intensities for T1 – T4 corresponds to 1.01, 2.37, 5.00, and 5.86 mm/hr, respectively. Considering that, our simulations from T2 – T4 likely represent the rainfall intensity conditions that may generate historical landslides between 1987 – 1996 (*5*).

For further analyses, we generate maps of the saturation ratios h/z [m/m] and h_w/z [m/m], as well as the three-dimensional components of the hydraulic gradient vector and hydraulic flux (i.e., the seepage vector and seepage flux) (Figs. 2-4, S4-11). The hydraulic gradient vector and flux, both vertically and horizontally, have been computed for each elementary volume across the entire computational domain, using the pressure head of the considered volume and its surrounding volumes. Specifically, we present results from the second cell in the soil above the soil-bedrock interface (subscript, *s*) and the second cell in the bedrock immediately below the soil-bedrock interface (subscript, *b*).

2.3 Slope stability model and landslide analyses

2.3.1 Multidimensional slope stability model modification

We adopt a multidimensional slope stability model coupled with a spectral search algorithm to predict shallow landslide occurrence, size, and location by testing the stability of clusters of adjacent grid cells (*26-28*). This model represents the landscape as a graph with vertices annotated with the driving forces contributed by individual soil columns and edges representing the forces that can develop between them. Using a spectral graph-theoretical approach similar to that proposed by Shi and Malik (*55*) in the context of image segmentation, the algorithm partitions the landscape graph by minimizing an objective function, in this case, the ratio of all the resistances acting on a cluster of cells and the total driving force contributed by those cells. This method produces a set of testable clusters of soil columns that may or may not fail as a shallow landslide. Predicted landslides are assumed to mobilize all soil in a cell, thus extending to the soil-bedrock boundary and encompassing an area equal to or larger than one cell. Our model evaluates only the triggering condition on the soil-bedrock boundary and does not simulate failure propagation, evacuation, runout, material removal, or recharge.

To define the forces and resistances acting on each cell and to test the stability of each cluster produced by the search algorithm, we modify a Mohr-Coulomb limit-equilibrium slope stability model that calculates the factor of safety as the ratio of driving to resisting forces on entire soil

clusters (*26*). This model assumes a rigid block failure on a plane parallel to the soil-bedrock interface. Using these assumptions, the model performs a force balance considering gravitationally induced driving forces, resistive forces resulting from friction and root cohesion with active, passive, and at-rest earth pressures acting on the boundaries of a landslide. Specifically, the model calculates resisting lateral forces R_l, resisting forces on downslope faces R_d, net driving forces on upslope faces R_u, basal shear resisting forces R_b, and basal driving forces F_b.

Here, we modify the basal resisting and driving force calculations of Milledge et al. (*26*) to include the effects of seepage flux between bedrock and soil, assuming these effects are negligible on the lateral, upslope, and downslope faces. We adopt the formulation of Iverson and Major (*56*) and calculate the magnitude of the hydraulic gradient or seepage vector, i_{mag} [m/m], as

$$i_{mag} = \sqrt{{i_x}^2 + {i_y}^2 + {i_z}^2}, \quad \text{(Eq. S4)}$$

where the components of the hydraulic gradient in the x, y, and z directions are i_x, i_y, and i_z [m/m], respectively, which are output from GEOtop 2.0. Seepage flux is calculated as the negative product of hydraulic conductivity and the seepage vector i. Similarly, we calculate the magnitude of seepage flux, f_{mag} [m/s], as

$$f_{mag} = \sqrt{{f_x}^2 + {f_y}^2 + {f_z}^2}, \quad \text{(Eq. S5)}$$

where the components of seepage fluxes in the x, y, and z directions are f_x, f_y, and f_z [m/m], respectively.

Next, we calculate the flux magnitude and angular orientation of the seepage vector relative to the interface of soil and bedrock layers (Fig. 1D). We calculate the orientation of the seepage vector λ (in degrees) in the downslope direction relative to the outwardly directed normal vector of the soil-bedrock boundary (*56*). A λ value of 0° – 90° indicates upward, exfiltrating seepage flow, while that of 90° – 180° indicates downward, infiltrating seepage flow, both oriented downslope. We define λ relative to the soil-bedrock boundary to be consistent with the direction of mass movement along the failure plane assumed by the slope stability model. Then, we calculate the seepage flux magnitude normal to the soil-bedrock boundary, f_{norm} [m/s], as the dot product of the seepage vector and the vector normal to the soil-bedrock boundary. f_{norm} is calculated as a multiplication of K, i_{mag}, and $\cos(\lambda)$. Thus, the positive or negative sign of f_{norm} indicates upward flux (e.g., exfiltration) or downward flux (e.g., infiltration) relative to the soil-bedrock boundary, respectively. We calculate f_{mag} and f_{norm} in the soil cell above (subscript, *s*) and the bedrock cell below (subscript, *b*) the soil-bedrock boundary. We then assume that the magnitude and orientation of the seepage vector in the soil above the soil-bedrock boundary will represent the flux critical for predicting failure along the soil-bedrock boundary.

We calculate a modified form of the slope-parallel basal driving force F_b [N] that considers seepage forces as

$$F_b = LWg[sin(\theta)(\rho_s z - \rho_w h) + \rho_w i_{mag} h sin(\lambda)], \quad \text{(Eq. S6)}$$

where plan view cell length is L [m], plan view cell width is W [m], g is gravitational acceleration [m/s^2], topographic slope is θ [degree], soil density is ρ_s [kg/m^3], vertical soil thickness is z [m], and water density is ρ_w [kg/m^3]. Similarly, we calculate the basal resisting force R_b [N], considering seepage forces as

$$R_b = LWg[cos(\theta)(\rho_s z - \rho_w h) - \rho_w i_{mag} h cos(\lambda)]tan(\phi) + C_b LW, \quad \text{(Eq. S7)}$$

where ϕ [deg.] is the soil friction angle and C_b [Pa] is the basal root cohesion.

The factor of safety *FoS* of an entire soil cluster is the sum of all resisting forces from all cells divided by the sum of all driving forces from all cells (*26-28*), defined as

$$FoS = \frac{\Sigma_{all} R_b + \Sigma_{lateral} R_l + \Sigma_{down} R_d - \Sigma_{up} R_u}{\Sigma_{all} F_b}, \quad \text{(Eq. S8).}$$

FoS includes lateral, downslope, and upslope resisting and driving forces at the edges of a cell cluster. The downslope resisting force R_d arises from passive earth pressure, whereas the upslope driving force R_u reflects active earth pressure. Lateral forces correspond to at-rest earth pressures. Soil friction and root cohesion are included along the basal surface as well as the upslope, downslope, and lateral boundaries. We do not consider water pressure on the upslope and downslope boundaries because pore-pressure distributions along these boundaries are complex and depend on both the failure geometry and the three-dimensional groundwater fluxes within each soil column. Refer to Milledge et al. (*26*) for R_l, R_d, and R_u derivations.

2.3.2 Model parameterization, outputs, and assessments

We apply the spectral clustering landslide model over the spatial domain of the benchmark site during specific times of interest. We consider the four different times from hydrologic modeling with GEOtop 2.0 (T1-T4, Fig. 1C). The output maps of h/z, i_{mag}, and λ from GEOtop 2.0 at these times are used as the hydrologic inputs for our landslide model.

We use modeled soil thickness, root cohesion, and soil properties as geologic inputs. The LiDAR DEM of the benchmark site is then the spatial domain of our landslide model. We set model parameters as $L = W = b = 2$ m, $\rho_s = 1600$ kg/m^3, $\rho_w = 1000$ kg/m^3, $g = 9.81$ m/s^2, $\phi = 40°$, following Bellugi et al. (*27, 28*), which are calibrated to the benchmark site (*6, 7, 9*). Here, we follow Milledge et al. (*26*) and Bellugi et al. (*27, 28*) to calculate C_b using previously established root cohesion from CB1 (*17*).

Applying these two models using field data, we use an integral method to solve for parameters for the average basal C_b [Pa] and lateral C_l [Pa] root cohesion (*26*). The parameters are calculated as

$$C_b = C_{r0} e^{-zj}, \quad \text{(Eq. S9)}$$

$$C_l = \frac{C_{r0}}{jz}(1 - e^{-zj}), \quad \text{(Eq. S10)}$$

where C_{r0} [Pa] is the maximum root cohesion at the surface, z [m] is the failure plane depth (equivalent to soil depth at each cell), and j [1/m] is an e-folding length scale. C_{r0} and j are obtained by fitting an exponential function to observations of root cohesion with depth reported by Montgomery et al. (*9*) for the CB1 scar. C_{r0} and j used in Bellugi et al. (*27, 28*), on which we base our landslide model, are 21,666 Pa and 4.96/m, respectively. Details of impacts from soil cohesion on landslide size and locations of shallow landslides were extensively examined in Bellugi et al. (*28*).

We modify a multidimensional slope stability model, coupled with a spectral search algorithm, to incorporate spatially variable seepage forces from our hydrologic model and predict the stability of clusters of adjacent grid cells (*26-28*). Our landslide model predicts many possible overlapping discrete landslides, but landslides are mutually exclusive, and only a small subset of these landslides will likely occur for any specific event. In addition, our model evaluates only the triggering condition and does not simulate evacuation or runout. Thus, a landslide cluster with $FoS < 1$ at an earlier time remains available to fail in subsequent time steps.

We first consider the entire population of predicted landslides to examine the full range of possible landslide locations and sizes to assess general patterns in a statistically representative manner. For each cell within the study area (i.e., 0.04 km^2) identified as being part of at least one potential discrete modeled landslide, we count the number of possible discrete landslides that include it (often, many configurations are possible for a given cell). Then, we divide this count by the total number of possible discrete landslides across the entire study area, thereby assigning each cell a relative frequency value. Consequently, the more discrete landslides a particular cell is involved in, the higher its relative frequency value. This relative frequency serves as a metric indicating each cell's likelihood of participating in potential landslides. A relative frequency greater than zero (i.e., non-zero relative frequency) thus indicates unstable areas where landslides are possible.

In addition, we apply a pruning method to all possible discrete landslides for selecting only one of these overlapping landslides most likely to fail. This selection method is applied because retaining all identified clusters would lead to an overestimation of the number of potential discrete landslides in any given storm. There are different methods of choosing which one to occur, which have been discussed in detail in previous studies (*27, 28*). Bellugi et al. (*27*) describe workflows to prune overlapping landslides by the most unstable cell cluster or that with the factor of safety *FoS* closest to but less than one. The former method assumes that the *FoS* can

be interpreted as a probability of failure (the most likely landslide has the minimum *FoS*), while the latter method assumes that the FoS is a proxy for the temporal evolution of landsliding (the most likely landslide is the first to cross the threshold of failure). It is unclear which pruning method best approximates landslides that occur in nature. Here, we show the locations and sizes of pruned landslides with the minimum *FoS* as the most likely to occur for a particular event.

For each discrete, overlapping, and unstable cell cluster, we calculate landslide size as the area of each cell cluster and topographic position as the median topographic index of the cell cluster. The $\log_{10}$ of the topographic index derived from the assumption of steady state subsurface flow by Dietrich et al. (*23*) is

$$Topographic\ index = log_{10}(\frac{A}{bsin(\theta)}), \quad \text{(Eq. S11)}$$

where the upstream contributing drainage area to a cell is A [m^2], channel width is b [m], and θ is the slope of soil-bedrock boundary. We assume the cell size as b. We present the median topographic index considering each cell in an unstable cluster. Low index values indicate landslides located in steep, non-convergent areas. High index values indicate locations farther down the valley axis where the drainage area is larger and the slope is gentler (*28*).

We assess landslide model performance by comparing seven observed landslides from the benchmark sites (*5*) with total unstable areas calculated from the non-zero relative frequency of possible discrete landslides (Dataset S2). We count the number of cells where a landslide is correctly predicted (true positives, TP) or correctly not predicted (true negatives, TN). A prediction error occurs when a landslide is incorrectly predicted where none occurred (false positive, FP) or when a landslide that occurred was not predicted (false negative, FN). These categories are used to calculate several performance metrics, such as accuracy, precision, recall, and the F1 score. Accuracy represents the correct labeling of any given location (accuracy = (TP + TN) / (TP + TN + FP + FN)). Precision indicates that a predicted landslide corresponds to a real landslide (precision = TP / (TP + FP)). Recall refers to an observed landslide that is successfully predicted (recall = TP / (TP + FN)). The F1 score, which balances precision and recall, is calculated as 2×(precision × recall) / (precision + recall), and provides a single measure of accuracy for cases where both false positives and false negatives are considered equally important.

Due to limitations in computational time and model domain size, we focus on simulating coupled three-dimensional transient hydrology and multidimensional slope stability models for a three-dimensional grid of a small benchmark catchment, characterized by ridge and valley topography. The main simulations incorporate five different CZ configurations and a single storm event. We modeled three-dimensional transient hydrology over a 2-month period and conducted multidimensional slope stability analyses at four selected timesteps (T1-T4) based on hydrologic outputs. However, with increasing computational power and the advancement of parallel computing, it will become feasible to simulate larger areas with more diverse CZ structures under multiple storm scenarios in the future.

3. Detailed hydrologic modeling results from the soil-bedrock boundary

The spatial patterns of h/z (pressure head/soil thickness) and h_w/z (height of water table/ soil thickness) are similar for any given CZ structure (Fig. S7A–J, Dataset S1). Positive magnitudes of $(h\text{-}h_w)/z$ (where pore pressure exceeds hydrostatic conditions) also show similar spatial patterns across all CZ structures (Fig. S7K–O). Areas located near channel heads and side hillslopes close to the main channel tend to have $(h\text{-}h_w)/z$ higher than those from the upper parts of hillslopes in all CZ structures.

To examine seepage flux contributions from bedrock to soil, we examine seepage flux magnitude and directions in soil above and bedrock below the soil-bedrock interface (Figs. S10, S11). To examine seepage flux contributions from weathered bedrock to soil, we analyze seepage vector orientation, flux magnitudes, and their normal components in the soil above and weathered bedrock below the soil-bedrock interface, both in map view (Fig. S10) and through cell-by-cell comparison (Fig. S11).

We find that seepage flux magnitude and directions in soil above and bedrock below the soil-bedrock interface show large spatial variations (Fig. S10). The cosines of the seepage vectors (Fig. S10A-E in soil and F-J in bedrock) show that, whereas the seepage vector is largely parallel to the soil-bedrock interface, the vector of seepage from underlying bedrock approaches normal to the interface along the hillslope border with the channel (Fig. S1A). Hence, this exfiltration pressure in the bedrock dissipates once it crosses into the soil. This is consistent with CB1 observations that did not show excessive pore pressures in the soil despite bedrock fracture controlled groundwater return flow to the soil. We find this pattern is most strongly expressed in the CZ(soil). Here, normal seepage vectors from the conductive, unweathered bedrock are forced into the soil due to channel boundary conditions, but the seepage vector in the overlying soil is essentially slope parallel (Fig. S10B, G).

Magnitude variations in f_{norm} largely reflect differences in contributing factors such as the hydraulic conductivities (K) of CZ layers, $\underline{i_{mag}}$, and $\cos(\lambda)$ (Fig. S10K-O (soil), P-T (bedrock), Dataset S1). In CZs without weathered bedrock, $f_{norm,b}$ is positive but of very low magnitude, reflecting low hydraulic conductivities in unweathered bedrock (Fig. S10P, Q). CZ(soil) tends to have small, positive $f_{norm,b}$ and $\cos(\lambda_b)$ close to one near the channel, which reflects groundwater flow exfiltrating from unweathered bedrock upward to the soil-bedrock interface. We find spatial variations of $f_{norm,s}$ in the soil of these CZs (Fig. S10K, L), which do not show any correspondence with $f_{norm,b}$ (Fig. S10P, Q). Considering the low magnitudes of infiltrating or exfiltrating groundwater flux from bedrock in these scenarios, such variations in $f_{norm,s}$ reflect variations in soil thickness, topography, or the soil-bedrock interface rather than influences from the underlying bedrock.

In CZ structures with weathered bedrock, bedrock contribution to soil flux depends on whether the underlying bedrock beneath the soil is weathered or unweathered (Fig. S10M–O, R–T, W–Y,

and S11D–F). Near channels and at the base of hillslopes, weathered bedrock thins, and unweathered bedrock is directly exposed beneath the soil. The lateral extent of unweathered bedrock beneath the soil at the base of hillslopes varies across these scenarios (Fig. 2C-E). In these areas with unweathered bedrock at soil-bedrock interface, f_{norm} and cos(λ) from soil and bedrock do not show good correspondence. $f_{norm,b}$ is mostly low, and cos(λ_b) shows large variations from ~ -0.5 to 1. In contrast, $f_{norm,s}$ shows a large variation, and cos(λ_s) is close to 0 (e.g., surface parallel) (Fig. S11D–F, M–O).

Over most of the landscape outside of hollows and the base of hillslopes, weathered bedrock is directly exposed below the soil (Fig. 2C-E). In areas where there is deep weathered bedrock beneath the soil, $f_{norm,s}$ is negative, documenting infiltration (Fig. S10M–O). For these areas underlain by weathered bedrock, we observe positive relationships between cos(λ_s) and cos(λ_b) and between $f_{norm,s}$ and $f_{norm,b}$ (Fig. S11D–F, J–L). This pattern is most strongly expressed in the seepage magnitude of topographically convergent areas. Near the upper part of the hillslope, cos(λ_s) tends to be negative, indicating infiltrating fluxes from soil to underlying bedrock. However, cos(λ_b) tends to be either negative or slightly positive, indicating infiltrating fluxes or exfiltrating fluxes from weathered bedrock (see Fig. S11R for a graphical explanation of the different orientations of the seepage vector fields). Near the channels and in the hollows (more topographically convergent areas), both cos(λ) and f_{norm} in saturated soil and bedrock are positive, indicating both exfiltrating seepage fluxes. In these convergent areas, $f_{mag,b}$ and $f_{mag,s}$ also show a positive relationship, closely following the predicted saturated hydraulic conductivity differences, ~ 0.23 (Fig. S11G–I). In some locations, high $f_{norm,b}$, high $f_{mag,b}$, and cos(λ_b) have no effect on those in soil (e.g., Fig. S11D).

We observe spatial variations in the magnitude of seepage flux normal to the soil boundary at the soil-bedrock boundary ($f_{norm,s}$) in both CZ(soil) and CZ(soil, lowK) without limited contributions from seepage fluxes originating from the unweathered bedrock (Fig. S10K, L). These variations suggest that local soil flux components, either upward or downward, can develop at the soil-bedrock interface due to water accumulation within the soil. Factors such as differences between the topographic surface and the soil-bedrock boundary, spatial variability in soil thickness, and lateral water accumulation due to convergence at the CZ interface could induce changes in seepage flux magnitude and orientation within the soil. These factors may cause deviations from parallel flow relative to the soil-bedrock interface, which produce similar $f_{norm,s}$ observed in the CZ scenarios with permeable bedrock. The variations in $f_{norm,s}$ observed in the CZ(soil, lowK), however, are different from other scenarios. This difference is potentially due to the strong contrast in saturated hydraulic conductivities and consequent sharp pressure gradients across the interface between bedrock and soil. This in turn can induce numerical instabilities in the computation of the horizontal component of the seepage flux between adjacent bedrock and soil cells, introducing uncertainty into some calculated values in the CZ(soil, lowK).

In summary, a cell-by-cell comparison of *h/z* across different CZ structures reveals considerable differences, particularly in local hillslope areas (Fig. S11A–C). We find positive relationships between $f_{norm,b}$ and $f_{norm,s}$ (S11D–F), along with locally high ratios of $f_{norm,b}/f_{mag,s}$ or $f_{norm,b}/f_{norm,s}$ in CZ structures with weathered bedrock (Fig. S10W–Y). This pattern indicates that the flux

contribution from weathered bedrock to soil above the soil-bedrock interface can be substantial, resulting in high $f_{norm,s}$ in specific areas and contributing to high seepage flux magnitude in soil above the soil-bedrock boundary ($f_{mag,s}$). For CZ(stress), areas with thin weathered bedrock in hollows and near the channel (i.e., topographic index > 3) exhibit $f_{norm,b}$ /$f_{norm,s}$ of ~ 0.44, higher than the expected 0.23 derived from the ratio between the saturated conductivity of soil and weathered bedrock. This increase in $f_{norm,b}$ is primarily attributed to cos(λ_b), which is ~ 1.85 times higher than cos(λ_s), indicating a greater upward seepage flux in weathered bedrock compared to soil (Fig. S11F, I, L).

Slope stability is influenced by these changes in *h/z*, seepage vector magnitude $i_{mag,s}$, and seepage vector orientations λ_s. Stark differences in *h/z* across all CZ structures indicate that the extent of deep CZ largely controls the spatial distribution of *h/z* in the upslope regions (Fig. 2). In addition to *h/z*, changes in the magnitudes and orientations of bedrock seepage exfiltration can influence those of soil flux, which can reduce the effective normal stress and increase shear stress of a soil landslide. A cell-by-cell comparison shows that seepage flux from the weathered bedrock may increase the components of soil normal to soil-bedrock interface by a small amount, largely through the changes in λ_s rather than $i_{mag,s}$ (Fig. S11, SI Appendix, section 3). Downslope thinning of deep CZ structures also adds more water into the soil, which can elevate pore pressures and contribute to destabilizing the soil mantle. Interestingly, these areas of localized groundwater exfiltration tend to produce similar sizes of predicted landslides, even in the later times of the model runs (e.g., T3 and T4 in CZ(stress), Fig. 5E).

4. Limitations on model assumptions and comparisons

Our integrated modeling approaches aim to assess the primary impact of variations in weathered bedrock on shallow landslides. However, these models cannot capture all processes operating over natural landscapes due to limited information and simplifying assumptions. First, as is common in all landslide models, the number of landslides predicted exceeds the observed (Fig. 2 U-AD). These overpredictions arise from a combination of insufficient knowledge of local subsurface conditions (e.g., soil thickness, root strength distribution, and local critical zone structure), inaccurate topographic data, and poor local rainfall records. Landslide mapping at the CB site was completed before a LiDAR survey was obtained. Hence, the size and location of landslides have some uncertainty. The scars were mapped in the field, rather than relying on less accurate mapping from aerial photographs. The soil production and transport models were locally calibrated (*7*). The effect of previous landslides on soil thickness and local stability are unknown, but likely mattered. The mapped landslides shown in the benchmark site (Fig. 1B) did not all occur with the CB1 storm, but rather preceded the storm, as discussed in Bellugi et al. (*28*).

Second, our models simulate bulk groundwater flow and landslide predictions using theoretical CZ structures without specifying fracture distributions or networks. The three-dimensional Richards equations in GEOtop 2.0 assume unsaturated flow through porous media without discretized fractures. We simulate the spatially varying boundary of weathered and unweathered bedrock with bulk hydraulic conductivities across two to seven orders of magnitude, depending

on the CZ structures used (Table S2). Previous work at CB1 found that fracture flow is important for groundwater fluxes and shallow landslide hazards (*8, 9, 15*). However, we currently do not have detailed information on the discrete fracture network at this site, nor the modeling capability to simulate preferential flow within fractured bedrock. We only simulate potential areas where groundwater infiltrates into uphill weathered bedrock and exfiltrates near channels, which is consistent with observations made in the field (*8, 15*). It is possible that non-Darcian fracture flow could produce significant local elevated seepage gradients at this site.

Nonetheless, we cannot model the exact location or magnitudes of exfiltrating seepage through bedrock heterogeneities like fractures. Our varying CZ structures aim to capture the bulk behavior of natural groundwater flow through fractures in weathered bedrock with spatial variations. We highlight the impact of topographically driven exfiltration from weathered bedrock on overlying soil h/z and subsequent landslides. Future work to include fracture flow based on spatial variations of fracture networks may allow for more accurate assessments of shallow landslide occurrence, size, and location. For example, previous studies have done pioneering fieldwork using Ground Penetrating Radar or drill core analysis to identify exfiltrating fracture systems in bedrock that likely contributed to landsliding (*57, 58*).

Third, we do not assert that our CZ structures encapsulate all natural variations in weathering at the benchmark site, nor do we claim that any specific CZ structure here is the best approximation of the actual CZ. The weathered bedrock layer in our model assumes no vertical gradation of hydraulic conductivity, which is different from slug tests that found variations of nearly five orders of magnitude within saprolite and fractured and weathered bedrock (*8, 15*). There are two conditions to consider when evaluating CZ structure best matches the field observations, keeping in mind that six of the landslides (CB2 failed in 1992) predated, by years, the storm that generated the CB1 failure. One is whether a landslide is predicted at the mapped landslide site, and the other is whether the timing of the predicted failure aligns with the observed event. CZ(RD9m) is the closest in meeting these criteria and in predicting the depth of the weathered, conductive layer under CB1. The matrix hydraulic conductivity in this pervasively oxidized and fractured layer may be higher than that in less fractured and partially oxidized bedrock below. Indeed, other hydrologic models focused on the CB1 landslide have been able to simulate discharge from weirs at the base of the CB1 hollow and reproduce measured discharge, assuming a weathered bedrock structure similar to CZ(RD4m) (*20*).

Fourth, our results regarding the influence of underlying bedrock on shallow landslides are derived from coupled hydrologic–stability models, and the parameters assigned to the CZ layers contain uncertainties and rely on simplified assumptions. Consequently, our findings depend on chosen parameter values and model configurations. Different parameter selections, alternative CZ layer geometries, rainfall histories, or hydrologic model setups—including initial and boundary conditions—may yield some difference in outcomes. For example, if failed material were removed during the simulation, the CZ (soil) scenario would likely not produce large landslides. In addition, we consider only downslope thinning of the deep CZ and use a single realization of soil-thickness variability. We also assume spatially uniform or simplified mechanical and hydrological properties within each CZ layer, including saturated hydraulic

conductivity, soil cohesion (derived from root strength), and friction angle. Variations in soil cohesion and soil thickness are known to significantly influence slope stability and landslide size distributions (*16, 28, 59, 60*), and may also contribute to observed clustering of landslides near hollows and channels. Bellugi et al. (*28*) specifically examined the impacts of variable root strength, pore pressure, and soil depth at this site, showing that increasing rainfall intensity or root cohesion generally increases landslide size and shifts failure locations downslope along hollow axes. More generally, Bellugi et al. (16) show that landslide size is controlled by the form, amplitude, and wavelength of spatial variability in material strength properties of hillslopes.

Lastly, our comparison of mapped and modeled discrete landslides has several limitations. Seven mapped landslides are used to compare the ranges of landslide occurrences, sizes, and locations of predicted landslides from our model. We acknowledge that five landslides did not occur during CB1 storms; only landslides in CB1 and CB2 occurred (9). CB2 had failed previously in February 1992 prior to 1996 storm (8). The February 1992 storm had an intensity of 3.3 mm/hr over 16 hours to 3.9 mm/hr over 9 hours, which was shorter duration, higher intensity rainfall than the 1996 event. Montgomery et al. (*5*) attribute the limited number of landslides in the 1996 storm (<15% of the total) to the prior failure of the most susceptible areas and to the regeneration of root strength 9 years after cutting. These effects are not captured in our simulations, which do not consider landslides prior to the 1996 storm (similar to Bellugi et al. (*28*)). This means a direct comparison between our simulated and observed landslides across all seven mapped landslides may not be appropriate. Thus, we present all possible landslides to illustrate the spatial concentration of possible landslides and use this population for assessments. We did not attempt to compare our predictions, based on a single storm, with each individually mapped landslide. Instead, we model landslides resulting from hydrology under different CZs to capture the first-order effect of spatially varying thicknesses of weathered bedrock on landslide occurrence, size, and location. The examples of non-overlapping possible discrete landslides, selected based on the minimum factor of safety (FoS_{min}), are shown to demonstrate examples of possible landslides (Figs. 2Z-AD, S6AO-BH).

Disclaimer: Any use of trade, firm, or product names is for descriptive purposes only and does not imply endorsement by the U.S. Government

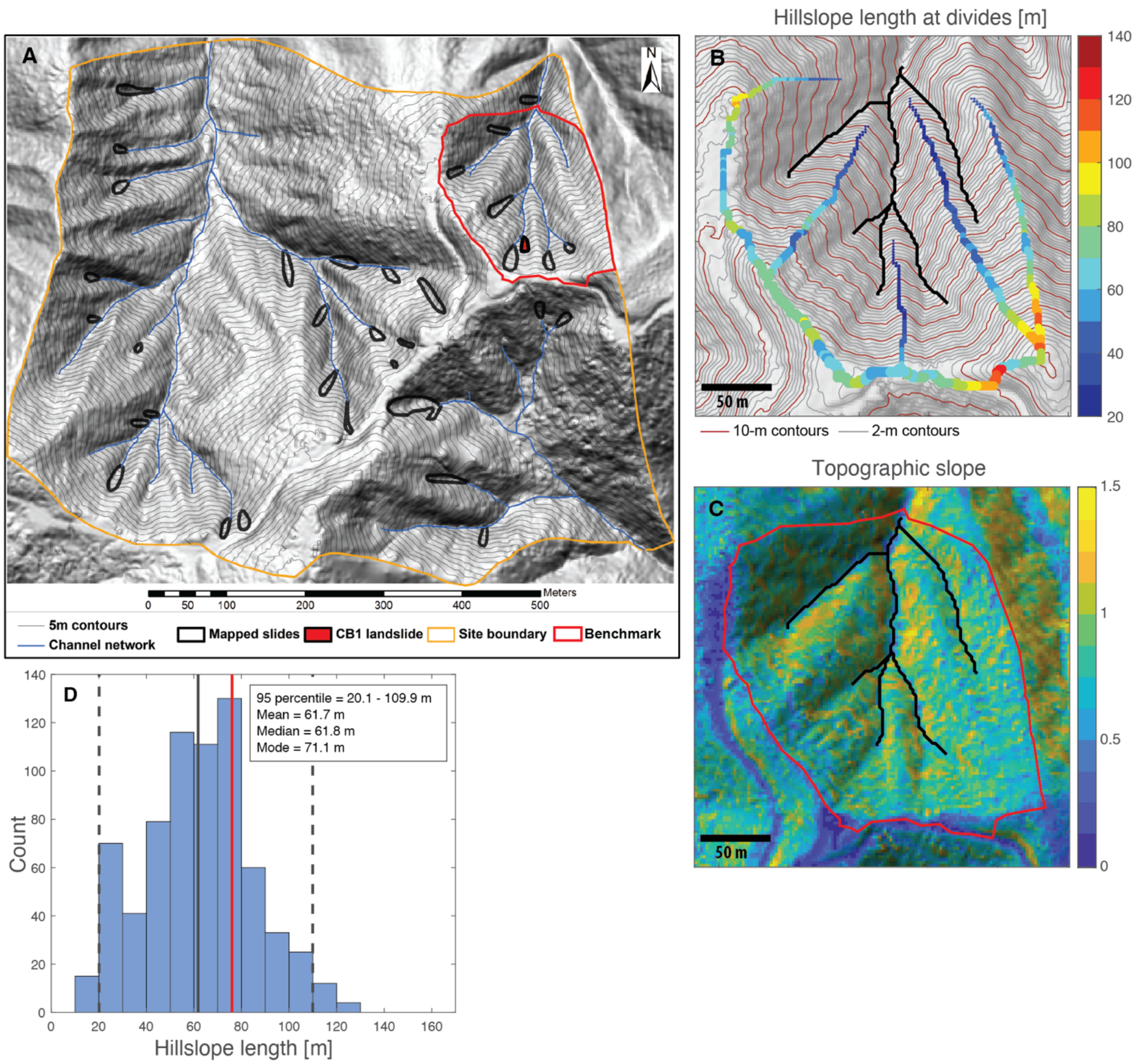

Fig. S1. (A) Shaded relief map of the Mettman Ridge study area with 5-m contours, the channel network, and the landslides mapped by Montgomery et al (*5*). The black polygons are landslides that occurred from 1987 and 1996 (*5*), and the CB1 landslide in red occurred in November 1996. Figure adapted from Bellugi et al. (*28*). (**B**) Hillslope lengths of the benchmark site were measured at drainage divides of Strahler order one (*40*). Black lines are channels of a 1500 m^2 drainage area threshold to calculate drainage divide locations that are used to model CZ structures. (**C**) Topographic slope as the steepest descent gradient (D8), the maximum downhill gradient (m/m) across a cell's eight neighbors. The boundary for our benchmark site is shown in a red polygon in A and C. (**D**) Histogram of hillslope length at drainage divides. The Y-axis is

the count of 2 m-resolution cells in a map of hillslope length that coincides with a divided segment within each 10 m-wide bin. The dashed line is a 95% confidence interval, the black line is the mean hillslope length, and the red line is a hillslope length of 76 m from Rempe and Dietrich (*30*).

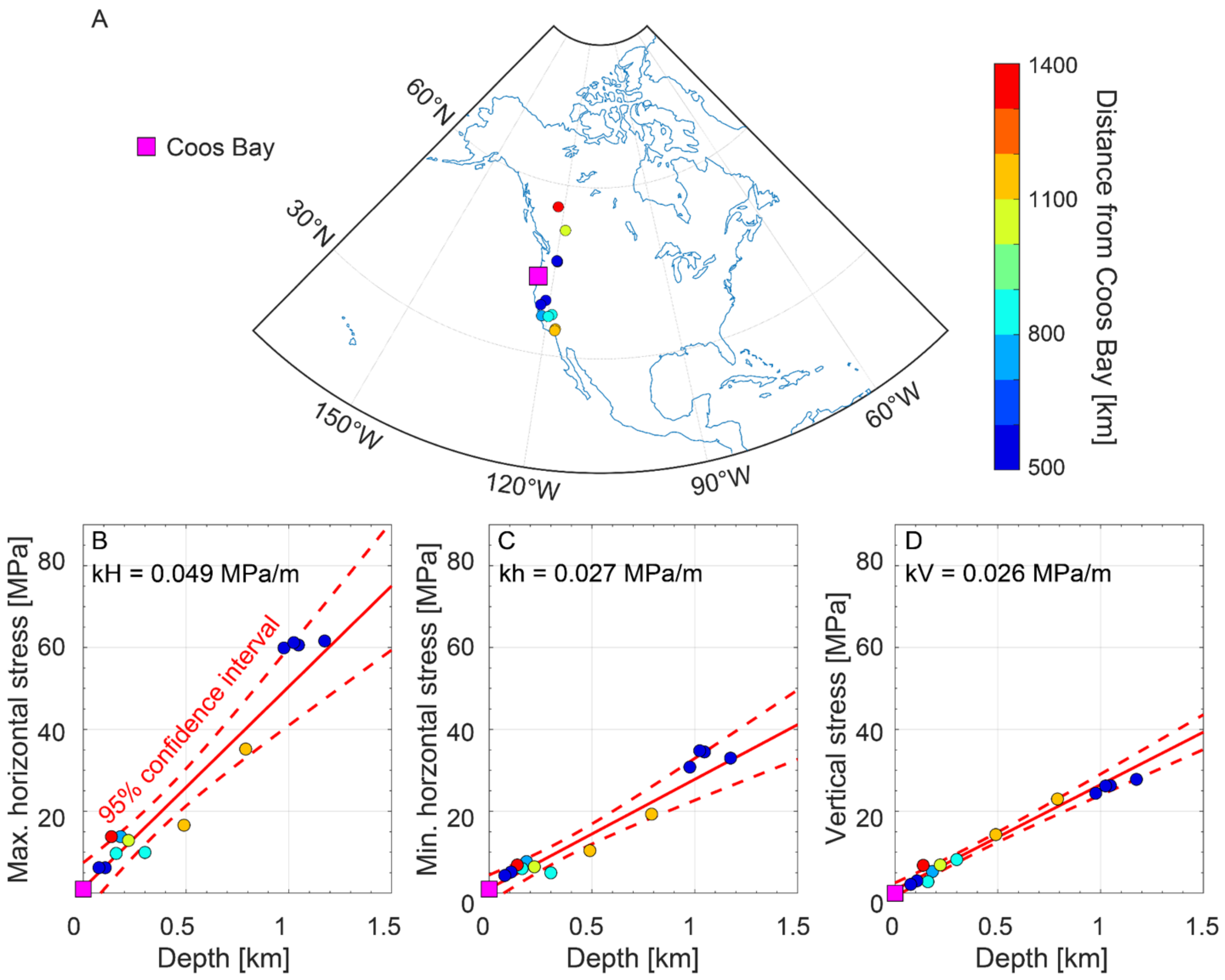

Fig. S2. (**A**) Location of in-situ stress magnitude and orientation from the World Stress Map 2016 (*33*) and other sources (*34, 35*) for a stress compilation applied at the CB1 site (*45*). (**B**) Depth versus maximum horizontal, (**C**) minimum horizontal, and (**D**) vertical stress magnitude [MPa], where kH, kh, and kV are the best-fit slope of the linear regression line in red that represents the gradient of stress magnitude with depth. All circular points are colored by distance from Coos Bay, Oregon, USA [km]. The pink square represents (A) the location of Coos Bay or (B–D) the stress magnitudes at the surface (y-intercept) of the benchmark site estimated from the regressions. Maximum horizontal, minimum horizontal, and vertical stress magnitude at the surface is 1 MPa, 1 MPa, and 0 MPa, respectively. Compressive stress is positive.

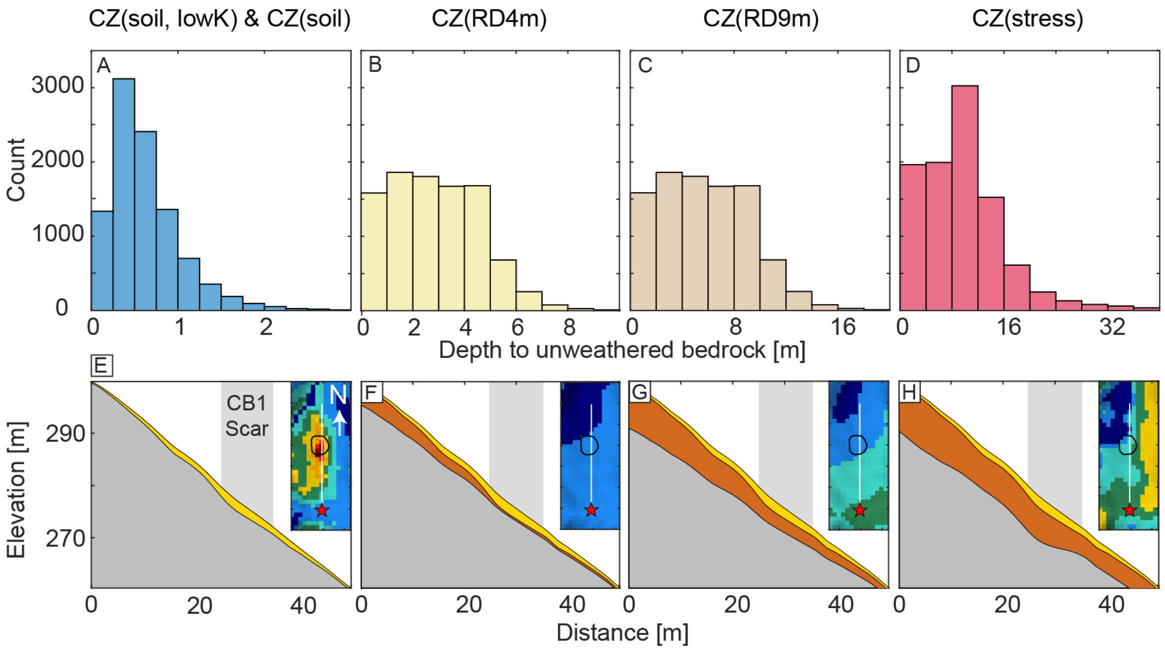

Fig. S3. Depth to unweathered bedrock. (**A – D**) Histogram of depth to unweathered bedrock by the total count of cells in each depth bin for the benchmark site. (**E – H**) Cross-section of critical zone structure along the white line in the inset, where yellow is soil, orange is weathered bedrock, and gray is unweathered bedrock. The extent of the CB1 scar is represented by a gray area. (Inset) Map of depth to unweathered bedrock using the same color scale as the corresponding critical zone model in **Fig. 2A – E**. The black outline is the CB1 landslide scar location, and the red star is the 35 m-deep CB1 borehole. The white line in **Fig. 2A** indicates the cross-section of **E – H**.

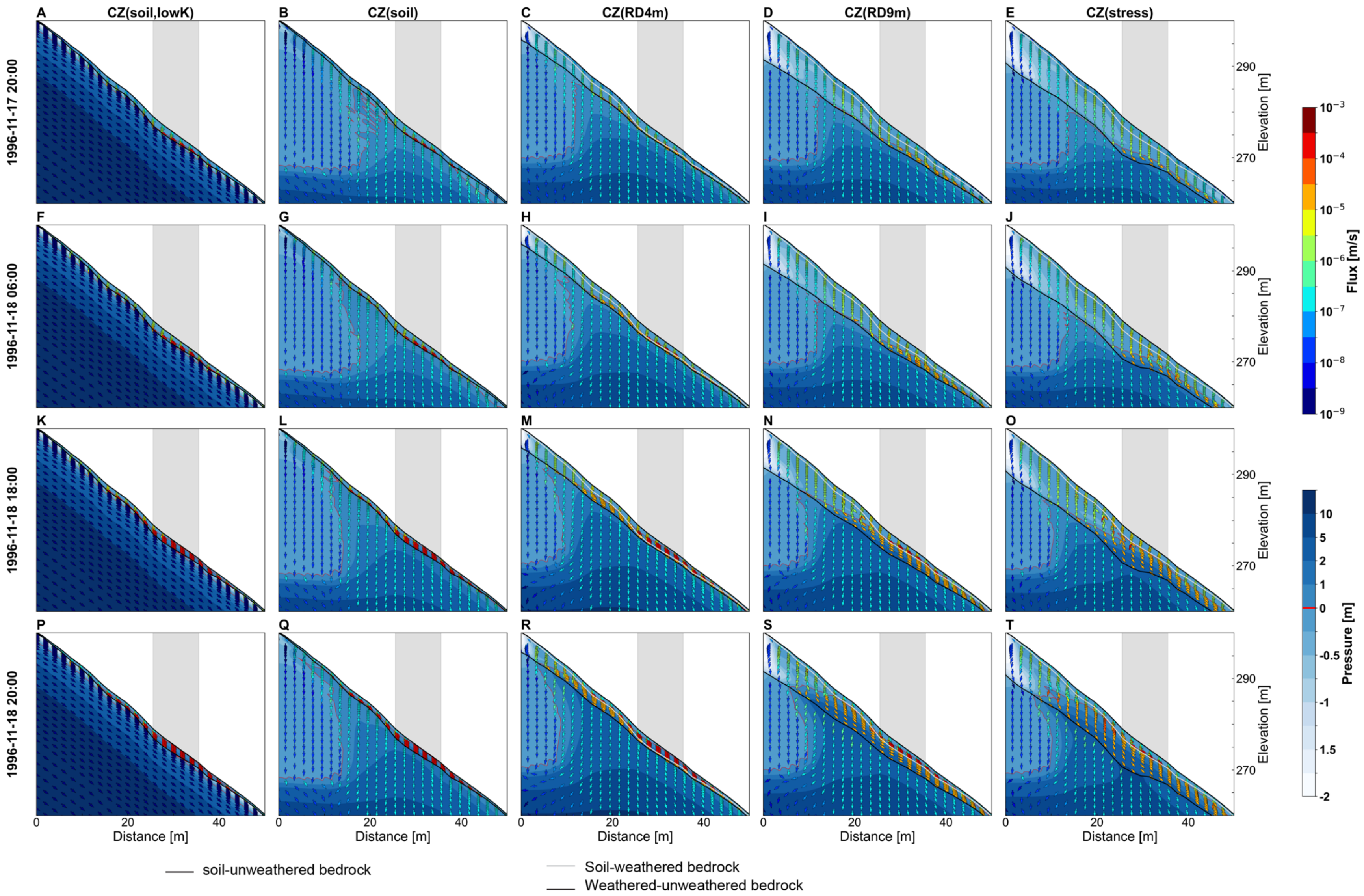

A
CZ(soil,lowK)
B
CZ(soil)
C
CZ(RD4m)
D
CZ(RD9m)
E
CZ(stress)
F
G
H
I
J
K
L
M
N
O
P
Q
R
S
T
1996-11-17 20:00
1996-11-18 06:00
1996-11-18 18:00
1996-11-18 20:00
Elevation [m]
290
270
Distance [m]
0
20
40
Flux [m/s]
10⁻³
10⁻⁴
10⁻⁵
10⁻⁶
10⁻⁷
10⁻⁸
10⁻⁹
Pressure [m]
10
5
2
1
0
-0.5
-1
1.5
-2
soil-unweathered bedrock
Soil-weathered bedrock
Weathered-unweathered bedrock

753

Fig. S4. **Groundwater flux magnitudes and directions for a north-south cross-section near CB1 for different times**. Different critical zone model scenario at (**A – E**) T1, 17 November 1996 20:00, (**F – J**) T2, 18 November 1996 06:00, (**K – O**) T3, 18 November 1996 18:00, and (**P – T**) T4, 18 November 1996 20:00. The direction and color of arrows represent the directions and magnitude of seepage fluxes, and pressure head values are shown as background. Black and gray solid lines show the soil-bedrock and weathered bedrock-unweathered bedrock boundaries, respectively. The extent of CB1 scar is represented by a gray area. Red line delineates the water table. Vertical scale is the same as horizontal, hence true slopes are shown. See Fig. S3 for the location of the cross section and the full extent of the model domain.

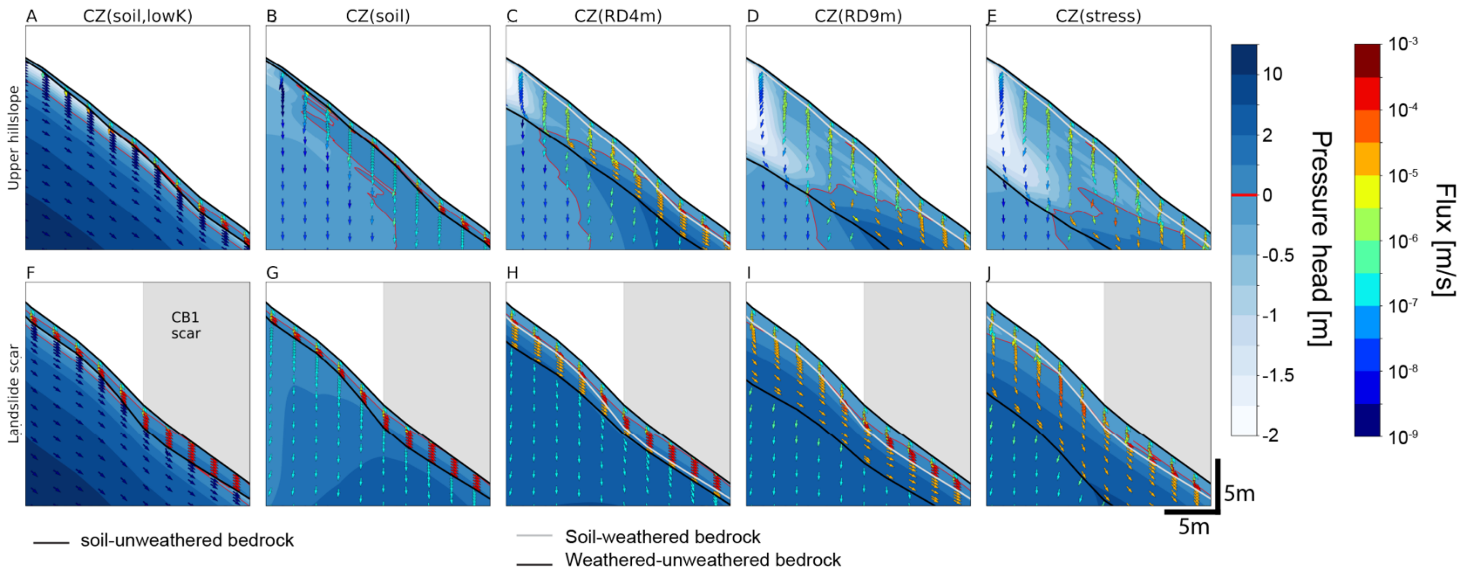

Fig. S5. Groundwater flux magnitudes and directions for two zoom-in sections within a north-south cross-section near CB1 at T4. Results are from (**A-E**) the upper hillslope near the ridge top and the (**F-J**) lower hillslope near the CB1 landslide scar. See Fig. 3E for the location of the zoom-in sections within the cross-section. (**A, F**) CZ(soil, lowK), (**B, G**) CZ(soil), (**C, H**) CZ(RD4m), (**D, I**) CZ(RD9m), and (**E, J**) CZ(stress) model scenario results at the time of the CB1 landslide (T4). The direction and color of the arrows represent the direction and magnitude of seepage fluxes, respectively. Pressure head values are shown in the background blue hue. The extent of the CB1 scar is represented by a gray area in F-J.

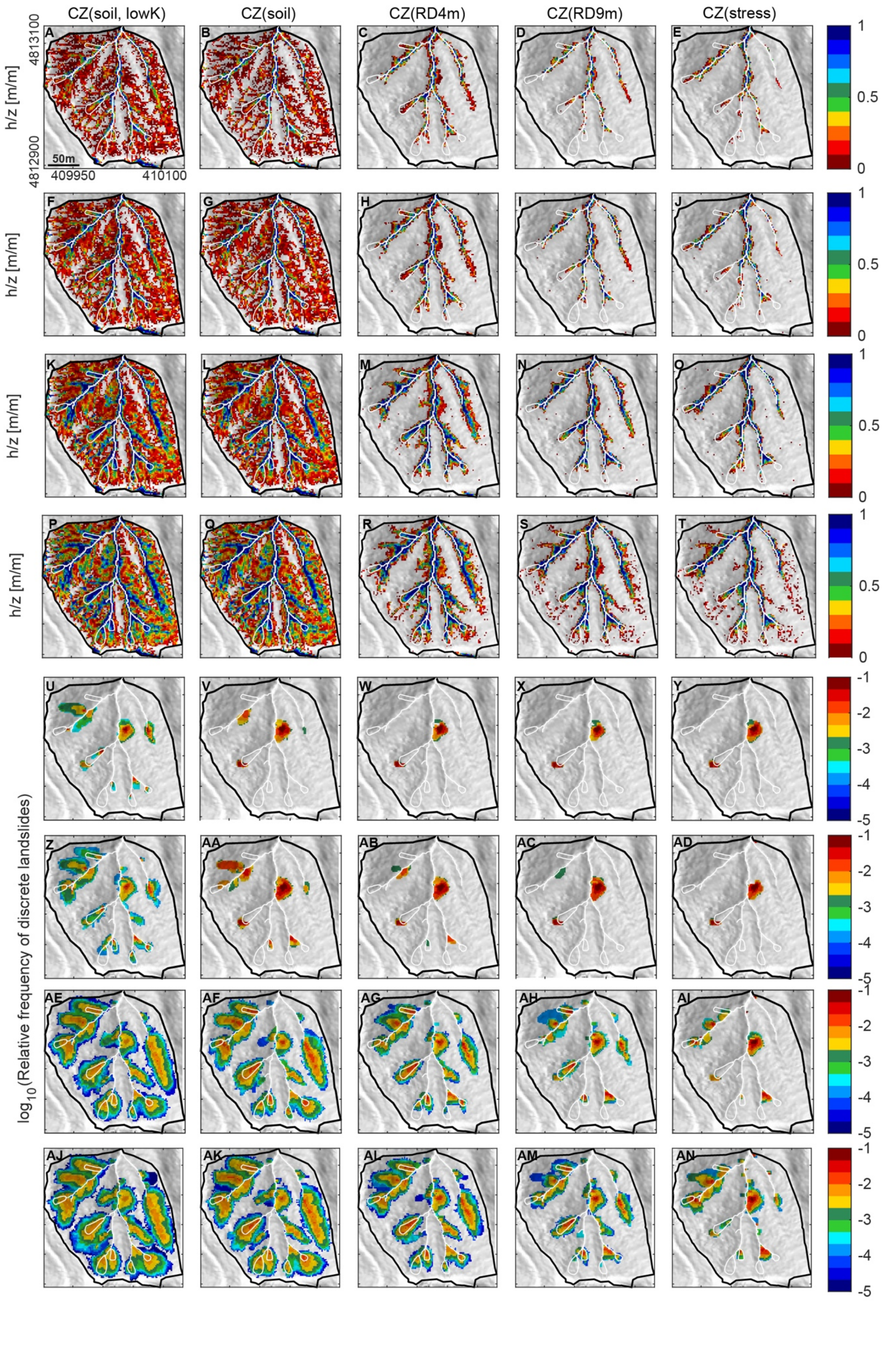
CZ(soil, lowK)
CZ(soil)
CZ(RD4m)
CZ(RD9m)
CZ(stress)
h/z [m/m]
4813100
4812900
50m
409950
410100
log10(Relative frequency of discrete landslides)
A
B
C
D
E
F
G
H
I
J
K
L
M
N
O
P
Q
R
S
T
U
V
W
X
Y
Z
AA
AB
AC
AD
AE
AF
AG
AH
AI
AJ
AK
AL
AM
AN
1
0.5
0
-1
-2
-3
-4
-5

771

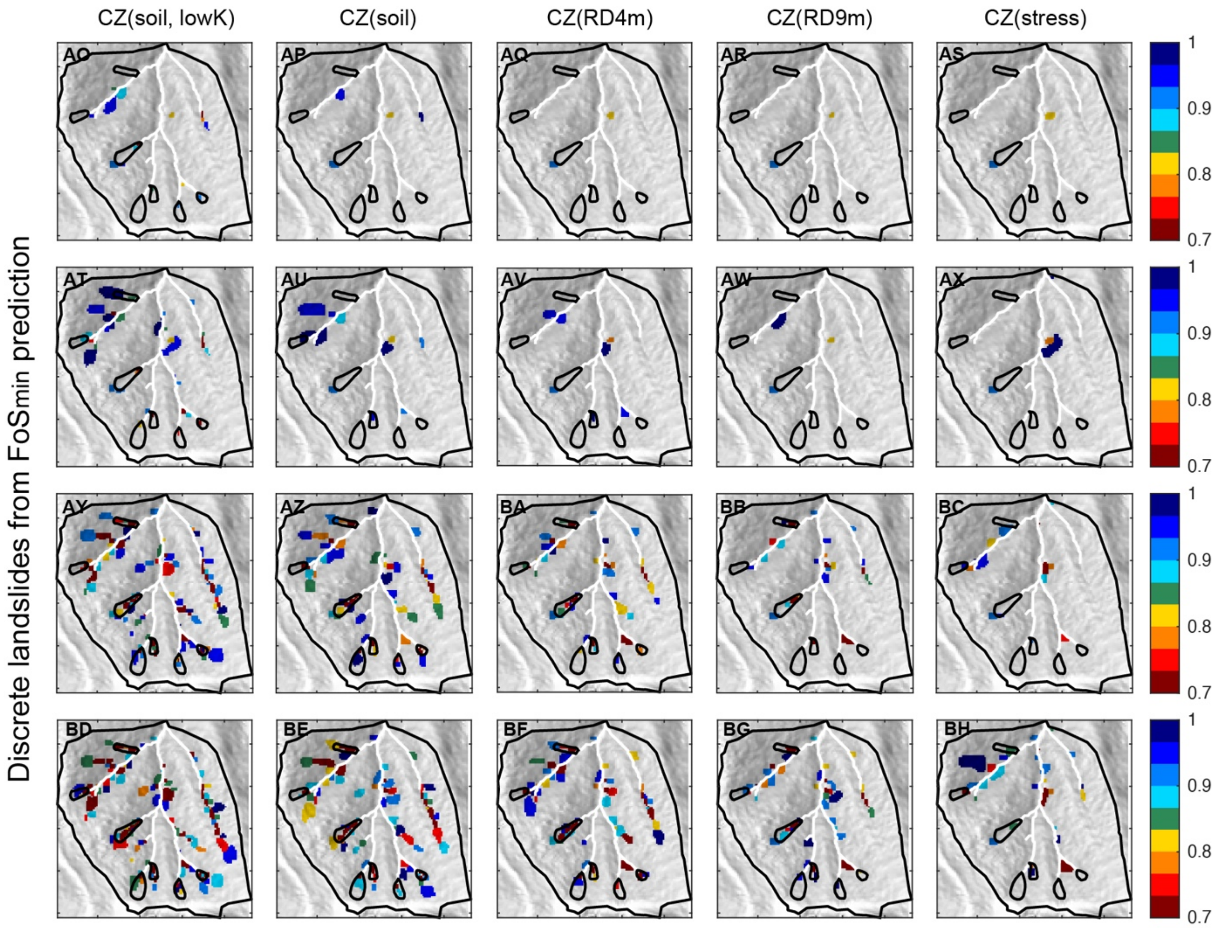

Fig. S6. Soil saturation and potential landslides for different critical zone models and times. (**A – T**) Soil saturation *h/z*, the ratio of pressure head at soil-bedrock boundary *h* and soil thickness *z* and (**U – AN**) $\log_{10}$ of the non-zero relative frequency of possible discrete landslides, calculated as the frequency of possible discrete landslides including a specific cell, normalized by the total number of possible landslides, and (**AO – BH**) non-overlapping possible discrete landslides, predicted by a minimum factor of safety (FoS_{min}). Results are for times at (**A – E, U – Y, AO–AS**) T1, (**F – J, Z – AD, AT–AX**), T2, (**K – O, AE-AI, AY–BC**) T3, and (**P – T, AJ – AN, BD – BH**) T4. This corresponds to 24, 14, 2, and 0 hours before the CB1 landslide. In CZ(soil, lowK) and CZ(soil), the upper parts of hillslopes remain unsaturated even in later times, as indicated by no colors. These areas typically have very thin soils (<0.15 m) and high moisture content-close to saturation, but not fully saturated. White lines = streams and landslides mapped between 1987 and 1996 (*5*). WGS84 UTM Zone 10N.

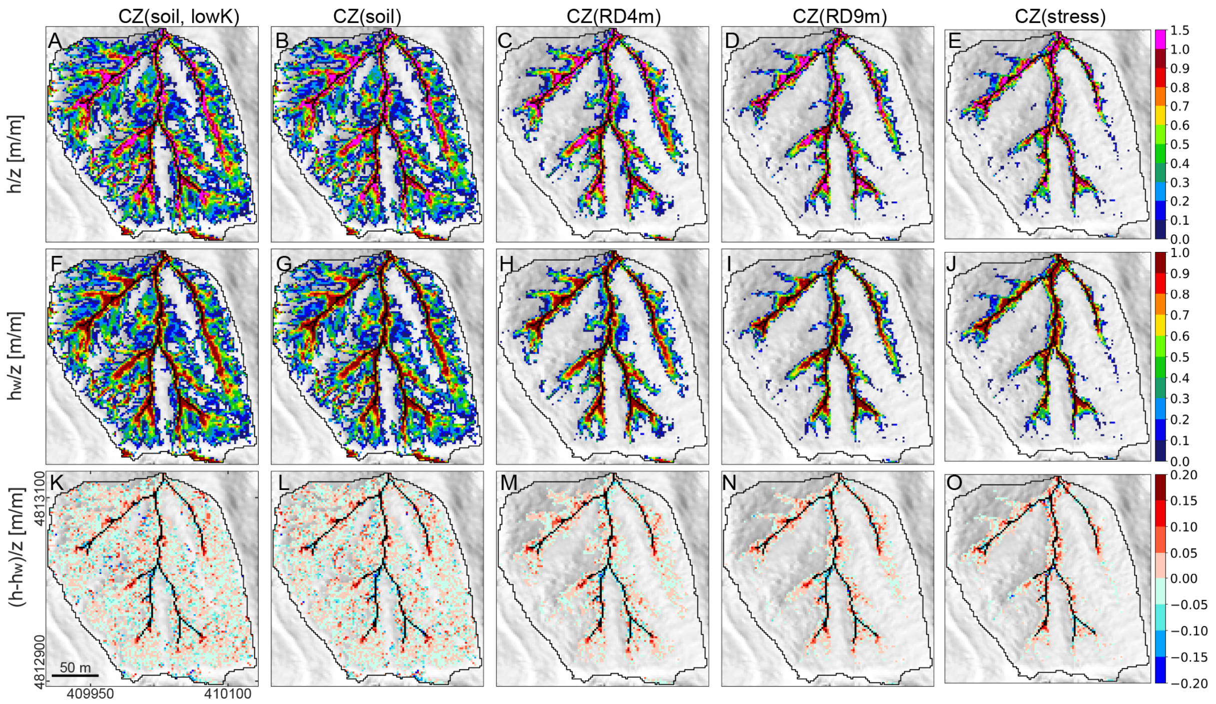

Fig. S7. Soil saturation calculation for different critical zone models at the time of CB1 landslide. (A – E) Soil saturation h/z, calculated as the ratio of pressure head at soil-bedrock boundary h to soil thickness z, (F – J) soil saturation h_w/z, calculated as the ratio of the height of water table (pressure head = 0) above the soil-bedrock boundary h_w to soil thickness z, and (K–O) $(h - h_w)/z$, which is the difference between h and h_w, normalized by z. A higher $(h - h_w)/z$ indicates areas with pore pressure higher than hydrostatic water pressure, likely due to water accumulation from flow convergence within the soil and the contribution from bedrock exfiltration (see SI Appendix, section 3, Dataset S1).

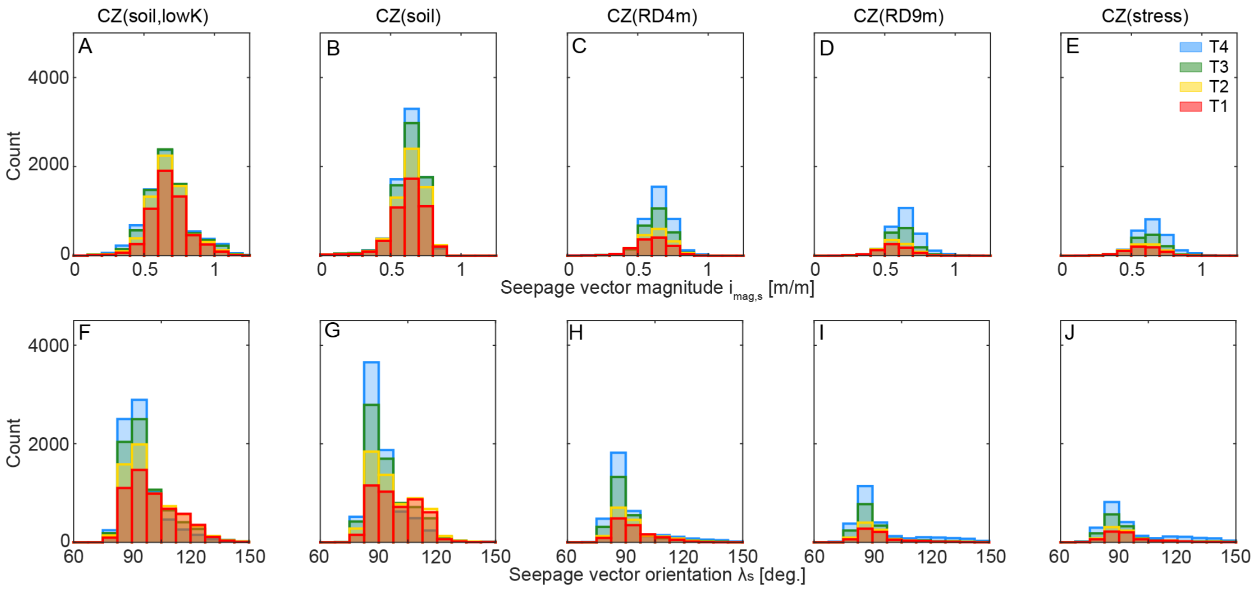

Fig. S8. Histograms of (**A – E**) seepage vector magnitude $i_{mag,s}$ in 0.1 m/m bins and (**F – J**) seepage vector orientation λ_s in 7.5° bins for different critical zone models. Colored histograms represent results from four different times during the CB1 storm. Seepage vectors are obtained from soil above the soil-bedrock boundary with h/z >0.

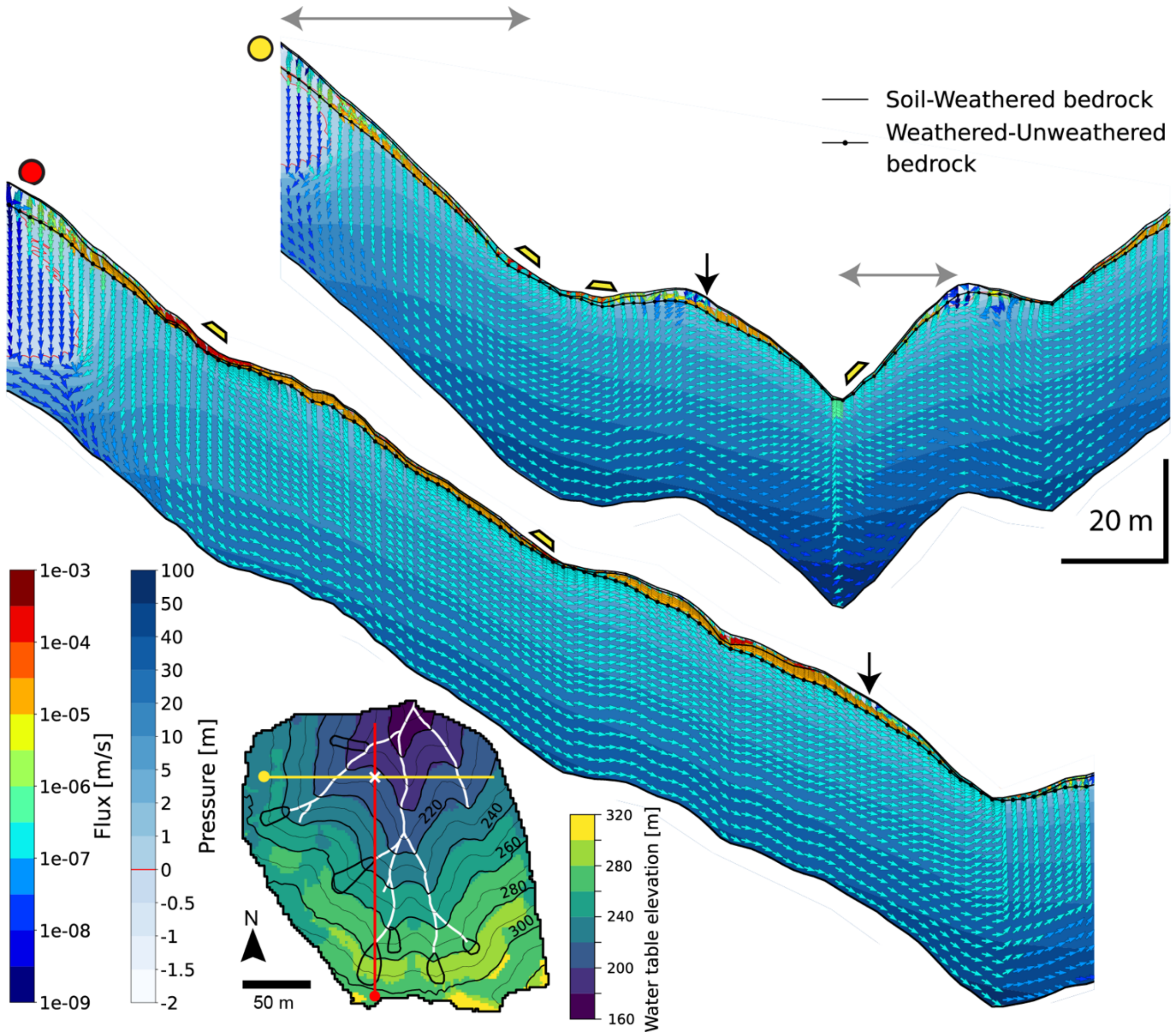

Fig. S9. Groundwater flux magnitudes and directions for cross-sections from CZ(RD4m) model. Results correspond to the time of the CB1 landslide event (T4). See the inset map for the locations of the south-to-north and east-to-west cross-sections, marked in red and yellow, respectively. The intersection point is indicated by a white cross on the inset map and black arrows on the cross-sections. The inset map color indicates water table elevation, while contours represent ground surface elevation. The direction and color of the arrows indicate the direction and magnitude of seepage fluxes, respectively, within the two-dimensional cross-sectional planes derived from three-dimensional model results. Pressure head values are shown in the background. Flux magnitudes and orientations within the weathered bedrock follow the topographic gradient due to the thin CZ layer. The locations of possible landslides, shown in Fig. 2W, are noted by trapezoidal symbols above the ground surface. Double arrows above the cross-section are orthogonal to the topographic slope.

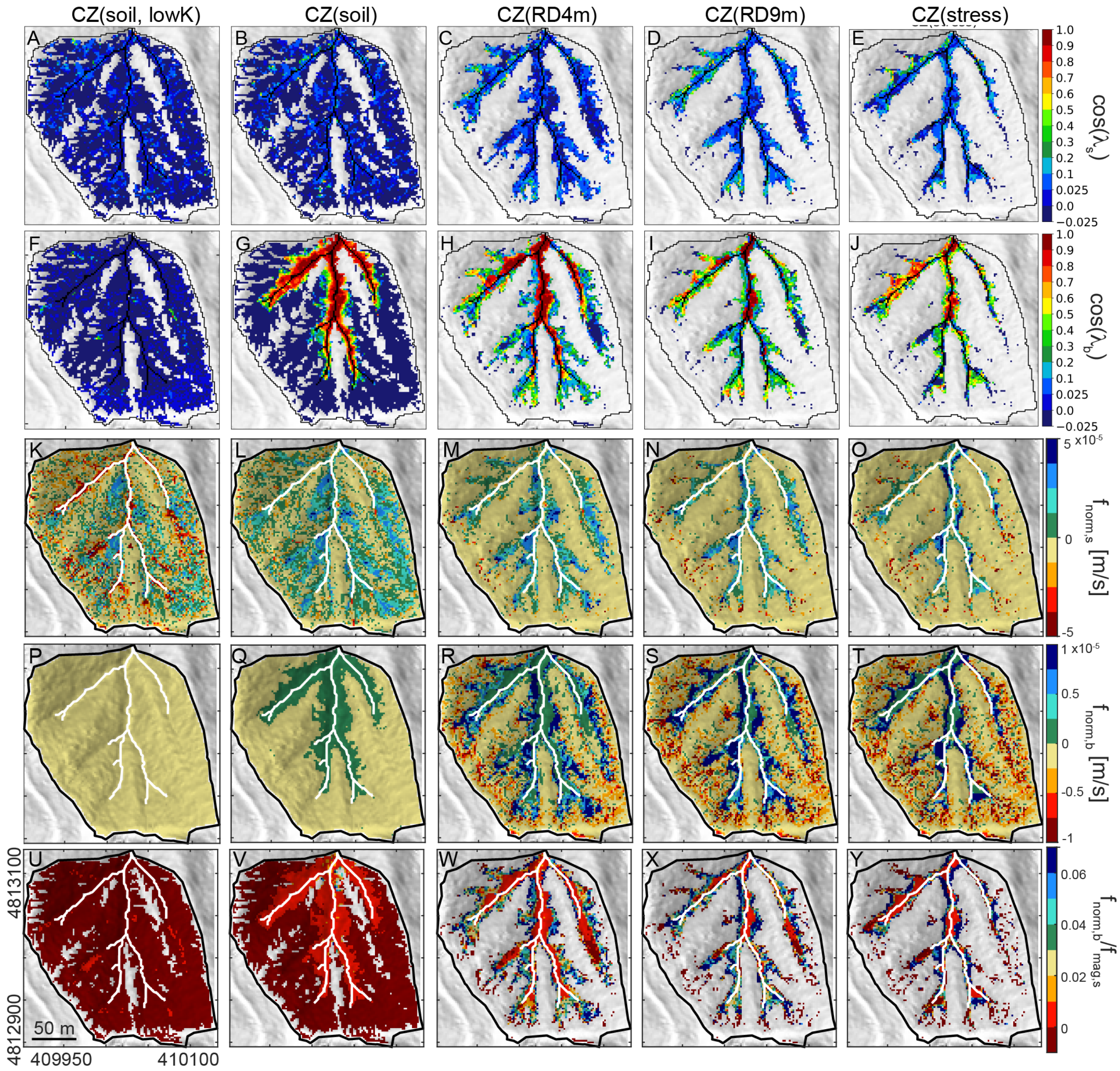

Fig. S10. Seepage vector orientation, flux magnitudes, and ratios below and above the soil-bedrock boundary for different critical zone models at the time of the CB1 landslide. Cosine of seepage vector orientation λ in (**A – E**) soil (λ_s) above and (**F – J**) bedrock (λ_b) below the soil-bedrock interface. A higher cos(λ) indicates a stronger vertically upward seepage component. Magnitude of seepage flux normal to the soil-bedrock boundary f_{norm} [m/s] in (**K – O**) soil ($f_{norm,s}$) above and (**P – T**) bedrock ($f_{norm,b}$) below the soil-bedrock boundary. (**U – Y**) The ratio of $f_{norm,b}$ [m/s] in the bedrock below and $f_{mag,s}$ in the soil above the soil-bedrock boundary. Ratios are presented only for cells with $h/z > 0$. Channel network shown as white lines. Benchmark site outline in black. Projection: WGS84 UTM Zone 10N. Note that the magnitude of saturated conductivity is 3.4 x 10^{-4} m/s for soil and 7.2 x 10^{-5} m/s for weathered bedrock.

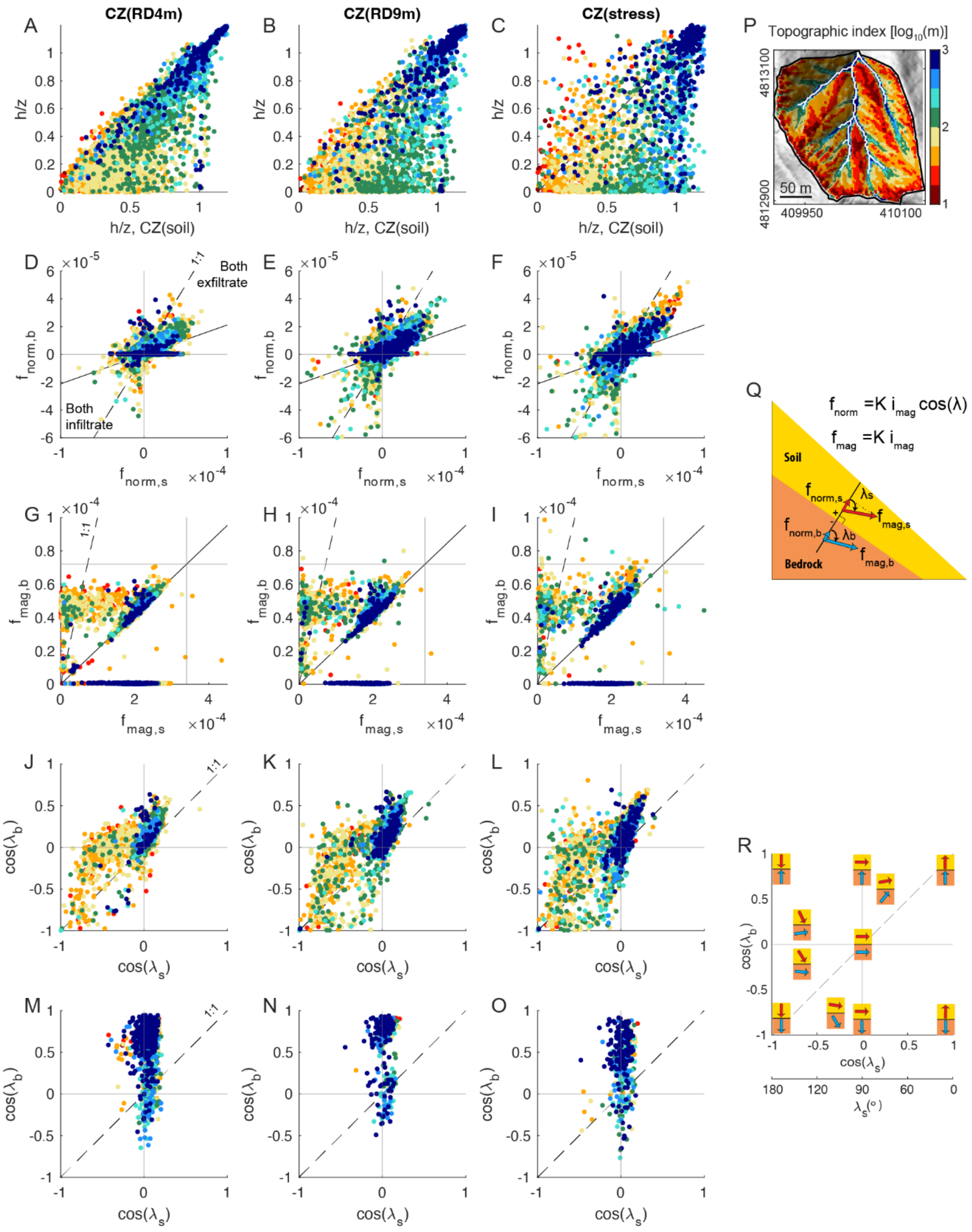

Fig. S11. Comparison between hydrologic properties from soil above and bedrock below the soil-bedrock interface from different CZ structures. Cell-by-cell comparison of **(A – C)** *h/z* from CZ scenarios compared to *h/z* from CZ(soil), and **(D – F)** $f_{norm,b}$ [m/s], (**G – I**) $f_{mag,b}$ [m/s], and (**J – O**) cos (λ_b) at the time of the CB1 landslide. The cells beneath the soil are divided by bedrock: (**J – L**) weathered bedrock and (**M – O**) unweathered bedrock. Schematic of critical

zone structure and hydrologic output variables that affect slope stability are shown in (**Q**). Subscripts *s* and *b* represent soil above and bedrock below the soil-bedrock interface, respectively. Results are (**A, D, G, J, M**) CZ(RD4m), (**B, E, H, K, N**) CZ(RD9m), and (**C, F, I, L, O**) CZ(stress). Results are used only for cells with $h/z > 0$. (**P**) Map of topographic index at the study site, with the channel network shown as white lines. Locations in (**P**) and the points in (**A – O)** are colored by corresponding values of the topographic index, a metric of topographic position (*61*) (Materials and Methods). Low index values in red are from steep and divergent areas, while high index values in blue are near valleys with gentle slopes and large drainage areas. The top right and bottom left quadrants in **D – F** and **J – O** indicate where cells in both soil and bedrock are exfiltrating and infiltrating, respectively. Examples of flux orientations for soil and bedrock relative to the horizontal surface in the corresponding plot space of $\cos(\lambda_b)$ and $\cos(\lambda_s)$ are shown in **R**. One-to-one lines are shown in the black dashed line in **D – O**, and lines with a slope of 0.23 are based on the ratio of saturated hydraulic conductivities shown in the black solid line in **D – I**. Saturated hydraulic conductivities of 3.4×10^{-4} m/s for soil and 7.2×10^{-5} m/s for weathered bedrock are shown in gray lines in **G – I.**

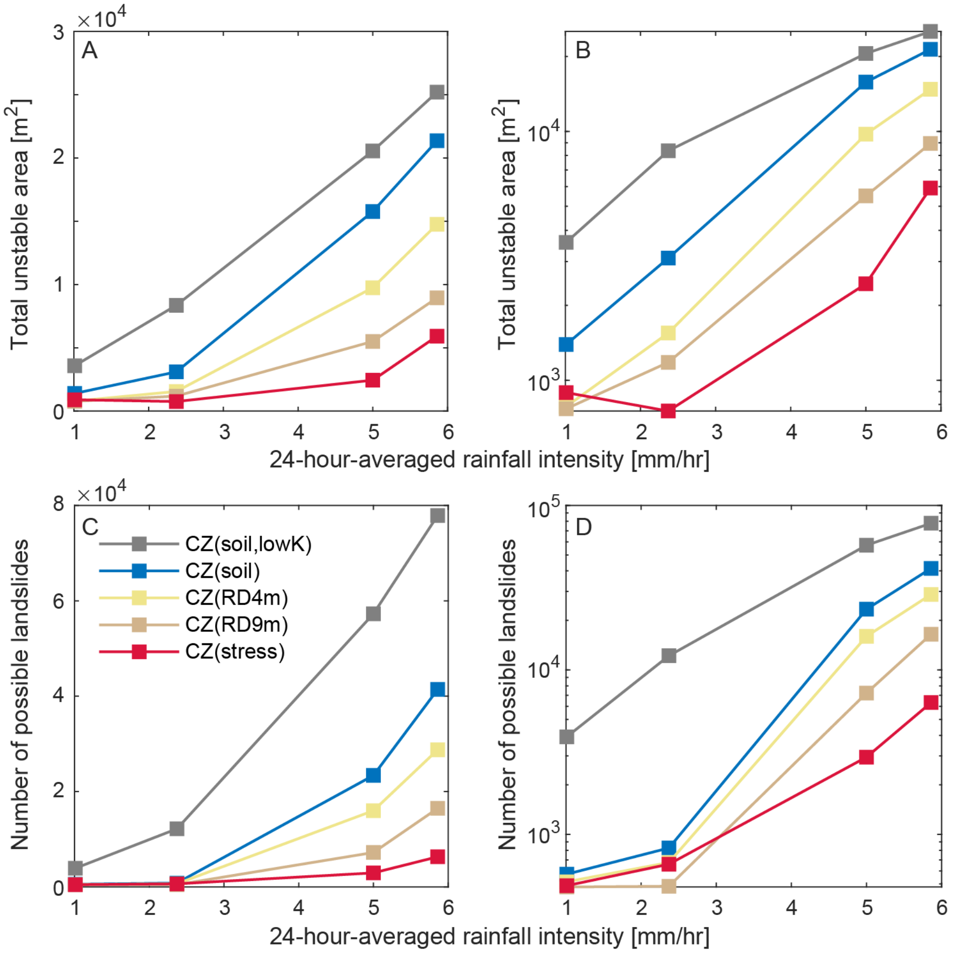

Fig. S12. Total number of predicted discrete landslides and total unstable area from CB1 storm simulations. (**A – B**) The total area of the benchmark site with a predicted non-zero frequency of possible discrete landslides and (**C – D**) the total number of possible discrete landslides that were identified as unstable clusters, many of which overlap in space. The simulation results for T1 to T4 are shown with the corresponding 24-hour-averaged rainfall intensities. Different critical zone model results are shown as different colored lines. **A** and **C** are in linear and **B** and **D** are in semi-log scales.

SI Tables

Table S1.

Critical zone column discretization for GEOtop 2.0.

Depth interval [m]	Vertical cell resolution [m]	Number of cells
0 - 2.5	0.05	50
2.5 - 3.5	0.1	10
3.5 - 5.5	0.2	10
5.5 - 23.5	0.3	60
23.5 - 40	0.5	33

Table S2.

Hydrologic properties of critical zone used for GEOtop 2.0.

Critical Zone Layer[a]	Residual water content [m^3/m^3]	Saturated water content [m^3/m^3]	Field capacity [m^3/m^3]	α [1/m][b]	n [-][b]	Specific storativity [1/m]	Porosity [m^3/m^3]	Saturated hydraulic conductivity [m/s]
Soil	0.20	0.50	0.3	0.018	2.8	$1x10^{-7}$	0.50	$3.4x10^{-4}$
Weathered bedrock	0.08	0.12	0.1	0.004	1.25	$1x10^{-7}$	0.15	$7.2x10^{-5}$
Unweathered bedrock	0.08	0.12	0.1	0.004	1.25	$1x10^{-7}$	0.12	CZ(soil, lowK) $5.0x10^{-12}$ or all else $5.0x10^{-7}$

[a] Data from previous studies (*3, 10, 11, 15, 20, 47*).
[b] α [1/m] is the scaling parameter related to the inverse of the air-entry pressure, and n [−] is a dimensionless measure of the pore-size distribution in van Genuchten model parameterization (SI Appendix, section 2).

Table S3.

Description and statistics of different critical zone models.

Model scenario name	Description[a]	Depth to unweathered bedrock [m]		Depth to unweathered bedrock at CB1 borehole location [m]	Percent landscape < 0.5 m-deep[c] [%]
		Mean ± 1 std	95% range[b]		
CZ(soil, lowK) & CZ(soil)	Modeled soil thickness, no weathered bedrock	0.6 ± 0.4	0.0 - 1.7	0.2	46.1

CZ(RD4m)	Modeled soil thickness, weathered bedrock based on Rempe and Dietrich (*30*)	2.9 ± 1.7	0.4 - 6.4	4.5	5.8
CZ(RD9m)	Modeled soil thickness, weathered bedrock based on Rempe and Dietrich (*30*)	5.8 ± 3.5	0.7 - 12.8	9	0.6
CZ(stress)	Modeled soil thickness, weathered bedrock based on topographic stress (*43*)	9.5 ± 6.3	0.4 - 25.9	9.6	3.1

[a] Detailed model description is provided in SI Appendix section 2.
[b] calculated as data range between 2.5 and 97.5 %
[c] calculated as the percentage of area within the benchmark site that has less than 0.5 m of the depth to the top of unweathered bedrock

Table S4. Description for variables in the main text

h	Pressure head at the soil-bedrock boundary	*m*
h_w	The height of water table (pressure head = 0) above the soil-bedrock boundary	*m*
z	Soil thickness	*m*
I	Rainfall intensity	*mm/hr*
D	Rainfall duration	*hours*
i	Seepage vector	*m/m*
i_{mag}	Magnitude of seepage vector	*m/m*
λ	Angular orientation	*deg.*
f	Seepage flux, same as specific discharge	*m/s*
f_{mag}	Magnitude of seepage flux	*m/s*
f_{norm}	Magnitude of seepage flux component normal to the soil-bedrock boundary	*m/s*
subscripts *s* and *b*	hydrologic quantities from soil above (s) and bedrock below (b) the soil-bedrock boundary, respectively	
$f_{mag,b}/f_{mag,s}$	the ratio of f_{mag} in bedrock to f_{mag} in soil	
$f_{norm,b}/f_{mag,s}$	the ratio of f_{norm} in bedrock to f_{mag} in soil	
K	hydraulic conductivity	m/s
FoS	Factor of Safety	
θ	slope of soil-bedrock boundary	deg.
A	drainage area	m^2
b	channel width	m

Dataset S1.

Hydrologic statistics for different critical zone models at different times of the CB1 storms measured at the soil-weathered bedrock boundary.

Dataset S2.

Landslide and slope stability statistics for different critical zone models at different times of the CB1 storms.

Excel file with Datasets S1 and S2.

SI References

1. J. J. Roering, J. W. Kirchner, W. E. Dietrich, Characterizing structural and lithologic controls on deep-seated landsliding: Implications for topographic relief and landscape evolution in the Oregon Coast Range, USA. *Geological Society of America Bulletin* **117**, 654-668 (2005).

2. S. P. Anderson, W. E. Dietrich, R. Torres, D. R. Montgomery, K. Loague, Concentration-discharge relationships in runoff from a steep, unchanneled catchment. *Water Resources Research* **33**, 211-225 (1997).

3. S. P. Anderson, W. E. Dietrich, G. H. Brimhall Jr, Weathering profiles, mass-balance analysis, and rates of solute loss: Linkages between weathering and erosion in a small, steep catchment. *Geological Society of America Bulletin* **114**, 1143-1158 (2002).

4. D. R. Montgomery, W. E. Dietrich, A physically based model for the topographic control on shallow landsliding. *Water resources research* **30**, 1153-1171 (1994).

5. D. R. Montgomery, K. M. Schmidt, H. M. Greenberg, W. E. Dietrich, Forest clearing and regional landsliding. *Geology* **28**, 311-314 (2000).

6. J. J. Roering, J. W. Kirchner, W. E. Dietrich, Evidence for nonlinear, diffusive sediment transport on hillslopes and implications for landscape morphology. *Water Resources Research* **35**, 853-870 (1999).

7. A. M. Heimsath, W. E. Dietrich, K. Nishiizumi, R. C. Finkel, Stochastic processes of soil production and transport: Erosion rates, topographic variation and cosmogenic nuclides in the Oregon Coast Range. *Earth Surface Processes and Landforms* **26**, 531-552 (2001).

8. D. R. Montgomery, W. E. Dietrich, J. T. Heffner, Piezometric response in shallow bedrock at CB1: Implications for runoff generation and landsliding. *Water Resources Research* **38**, (2002).

9. D. R. Montgomery, K. M. Schmidt, W. E. Dietrich, J. McKean, Instrumental record of debris flow initiation during natural rainfall: Implications for modeling slope stability. *Journal of Geophysical Research: Earth Surface* **114**, (2009).

10. R. Torres, W. E. Dietrich, D. R. Montgomery, S. P. Anderson, K. Loague, Unsaturated zone processes and the hydrologic response of a steep, unchanneled catchment. *Water Resources Research* **34**, 1865-1879 (1998).

11. B. A. Ebel *et al.*, Near-surface hydrologic response for a steep, unchanneled catchment near Coos Bay, Oregon: 1. Sprinkling experiments. *American Journal of Science* **307**, 678-708 (2007).

12. W. E. Dietrich *et al.*, Geomorphic transport laws for predicting landscape form and dynamics. *Geophysical Monograph-American Geophysical Union* **135**, 103-132 (2003).

13. J. D. Stock, W. E. Dietrich, Erosion of steepland valleys by debris flows. *Geological Society of America Bulletin* **118**, 1125-1148 (2006).

14. E. M. Baldwin *et al.*, Geology and mineral resources of Coos County. *Oregon: State of Oregon Department of Geology and Mineral Industries Bulletin* **80**, 82 (1973).

15. D. R. Montgomery *et al.*, Hydrologic response of a steep, unchanneled valley to natural and applied rainfall. *Water Resources Research* **33**, 91-109 (1997).

16. D. G. Bellugi, D. G. Milledge, K. M. Cuffey, W. E. Dietrich, L. G. Larsen, Controls on the size distributions of shallow landslides. *Proceedings of the National Academy of Sciences* **118**, e2021855118 (2021).

17. K. Schmidt *et al.*, The variability of root cohesion as an influence on shallow landslide susceptibility in the Oregon Coast Range. *Canadian Geotechnical Journal* **38**, 995-1024 (2001).

18. C. Cronkite-Ratcliff, K. M. Schmidt, C. Wirion, Comparing root cohesion estimates from three models at a shallow landslide in the Oregon Coast Range. *GeoHazards* **3**, 428-451 (2022).

19. D. R. Montgomery, W. E. Dietrich, Runoff generation in a steep, soil-mantled landscape. *Water Resources Research* **38**, (2002).

20. B. A. Ebel *et al.*, Near-surface hydrologic response for a steep, unchanneled catchment near Coos Bay, Oregon: 2. Physics-based simulations. *American Journal of Science* **307**, 709-748 (2007).

21. B. A. Ebel, K. Loague, D. R. Montgomery, W. E. Dietrich, Physics-based continuous simulation of long-term near-surface hydrologic response for the Coos Bay experimental catchment. *Water Resources Research* **44**, (2008).

22. B. A. Ebel, K. Loague, R. I. Borja, The impacts of hysteresis on variably saturated hydrologic response and slope failure. *Environmental Earth Sciences* **61**, 1215-1225 (2010).

23. W. E. Dietrich, D. Bellugi, R. R. De Asua, Validation of the shallow landslide model, SHALSTAB, for forest management. *Water science and Application* **2**, 195-227 (2001).

24. R. Rosso, M. C. Rulli, G. Vannucchi, A physically based model for the hydrologic control on shallow landsliding. *Water Resources Research* **42**, (2006).

25. R. I. Borja, J. A. White, Continuum deformation and stability analyses of a steep hillside slope under rainfall infiltration. *Acta Geotechnica* **5**, 1-14 (2010).

26. D. G. Milledge, D. Bellugi, J. A. McKean, A. L. Densmore, W. E. Dietrich, A multidimensional stability model for predicting shallow landslide size and shape across landscapes. *Journal of Geophysical Research: Earth Surface* **119**, 2481-2504 (2014).

27. D. Bellugi *et al.*, A spectral clustering search algorithm for predicting shallow landslide size and location. *Journal of Geophysical Research: Earth Surface* **120**, 300-324 (2015).

28. D. Bellugi, D. G. Milledge, W. E. Dietrich, J. T. Perron, J. McKean, Predicting shallow landslide size and location across a natural landscape: Application of a spectral clustering search algorithm. *Journal of Geophysical Research: Earth Surface* **120**, 2552-2585 (2015).

29. S. P. Anderson *et al.*, Subsurface flow paths in a steep, unchanneled catchment. *Water Resources Research* **33**, 2637-2653 (1997).

30. D. M. Rempe, W. E. Dietrich, A bottom-up control on fresh-bedrock topography under landscapes. *Proceedings of the National Academy of Sciences* **111**, 6576-6581 (2014).

31. R. A. Freeze, J. A. Cherry, *Groundwater* Englewood Cliffs, NJ (Prentice-Hall, 1979), vol. 176, pp. 604.

32. J. St. Clair *et al.*, Geophysical imaging reveals topographic stress control of bedrock weathering. *Science* **350**, 534-538 (2015).

33. O. Heidbach *et al.*, The World Stress Map database release 2016: Crustal stress pattern across scales. *Tectonophysics* **744**, 484-498 (2018).

34. E. N. Lindner, J. A. Halpern, in *International Journal of Rock Mechanics and Mining Sciences & Geomechanics Abstracts*. (Elsevier, 1978), vol. 15, pp. 183-203.

35. E. T. Brown, E. Hoek, in *International Journal of Rock Mechanics and Mining Sciences & Geomechanics Abstracts*. (Pergamon, 1978), vol. 15, pp. 211-215.

36. W. E. Dietrich, R. Reiss, M. L. Hsu, D. R. Montgomery, A process-based model for colluvial soil depth and shallow landsliding using digital elevation data. *Hydrological processes* **9**, 383-400 (1995).

37. S. L. Reneau, W. E. Dietrich, Depositional history of hollows on steep hillslopes, coastal Oregon and Washington. *National Geographic Research* **6**, 220-230 (1991).

38. S. Orlandini, G. Moretti, M. Franchini, B. Aldighieri, B. Testa, Path-based methods for the determination of nondispersive drainage directions in grid-based digital elevation models. *Water Resources Research* **39**, (2003).

39. J. Bear, *Dynamics of fluids in porous media*. (Dover, New York, 1988).

40. W. Schwanghart, D. Scherler, TopoToolbox 2–MATLAB-based software for topographic analysis and modeling in Earth surface sciences. *Earth Surface Dynamics* **2**, 1-7 (2014).

41. W. Dietrich, T. Dunne, Sediment budget for a small catchment in a mountainous terrain. (1978).

42. W. E. Dietrich, T. Dunne, The channel head. *Channel network hydrology* **799**, 175-219 (1993).

43. S. Moon, J. Perron, S. Martel, W. Holbrook, J. St. Clair, A model of three-dimensional topographic stresses with implications for bedrock fractures, surface processes, and landscape evolution. *Journal of Geophysical Research: Earth Surface* **122**, 823-846 (2017).

44. D. J. Miller, T. Dunne, Topographic perturbations of regional stresses and consequent bedrock fracturing. *Journal of Geophysical Research: Solid Earth* **101**, 25523-25536 (1996).

45. J. T. Higa, Multi-scale investigations on the impacts of geologic fractures. University of California, Los Angeles, (2023).

46. A. L. Thomas, POLY3D: A three-dimensional, polygonal element, displacement discontinuity boundary element computer program with applications to fractures, faults, and cavities in the earth's crust. Stanford University, (1993).

47. S. Endrizzi, S. Gruber, M. Dall'Amico, R. Rigon, GEOtop 2.0: simulating the combined energy and water balance at and below the land surface accounting for soil freezing, snow cover and terrain effects. *Geoscientific Model Development* **7**, 2831-2857 (2014).

48. S. Panday, P. S. Huyakorn, A fully coupled physically-based spatially-distributed model for evaluating surface/subsurface flow. *Advances in Water Resources* **27**, 361-382 (2004).

49. L. A. Richards, Capillary conduction of liquids through porous mediums. *Physics* **1**, 318-333 (1931).

50. M. T. Van Genuchten, A closed-form equation for predicting the hydraulic conductivity of unsaturated soils. *Soil Science Society of America Journal* **44**, 892-898 (1980).

51. Y. Mualem, A new model for predicting the hydraulic conductivity of unsaturated porous media. *Water Resources Research* **12**, 513-522 (1976).

52. G. Formetta, G. Capparelli, P. Versace, Evaluating performance of simplified physically based models for shallow landslide susceptibility. *Hydrology and Earth System Sciences* **20**, 4585-4603 (2016).

53. S. Kollet *et al.*, The integrated hydrologic model intercomparison project, IH-MIP2: A second set of benchmark results to diagnose integrated hydrology and feedbacks. *Water Resources Research* **53**, 867-890 (2017).

54. Y.-S. Wu, C. Haukwa, G. S. Bodvarsson, A site-scale model for fluid and heat flow in the unsaturated zone of Yucca Mountain, Nevada. *Journal of Contaminant Hydrology* **38**, 185-215 (1999).

55. J. Shi, J. Malik, Normalized cuts and image segmentation. *IEEE Transactions on pattern analysis and machine intelligence* **22**, 888-905 (2000).

56. R. M. Iverson, J. J. Major, Groundwater seepage vectors and the potential for hillslope failure and debris flow mobilization. *Water Resources Research* **22**, 1543-1548 (1986).

57. A. C. da Silva, I. Resende, R. C. da Costa, R. E. S. Uagoda, A. d. S. Avelar, Geophysical for granitic joint patern and subsurface hydrology related to slope instability. *Journal of Applied Geophysics* **199**, 104607 (2022).

58. A. Atwood *et al.*, Importance of subsurface water for hydrological response during storms in a post-wildfire bedrock landscape. *Nature Communications* **14**, 3814 (2023).

59. L. Fan, P. Lehmann, D. Or, Effects of soil spatial variability at the hillslope and catchment scales on characteristics of rainfall-induced landslides. *Water Resources Research* **52**, 1781-1799 (2016).

60. J. P. Prancevic, M. P. Lamb, B. W. McArdell, C. Rickli, J. W. Kirchner, Decreasing landslide erosion on steeper slopes in soil-mantled landscapes. *Geophysical Research Letters* **47**, e2020GL087505 (2020).

61. W. E. Dietrich, C. J. Wilson, D. R. Montgomery, J. McKean, R. Bauer, Erosion thresholds and land surface morphology. *Geology* **20**, 675-679 (1992).
